# MDRL lncRNA Regulates the Processing of miR-484 Primary Transcript by Targeting miR-361

**DOI:** 10.1371/journal.pgen.1004467

**Published:** 2014-07-24

**Authors:** Kun Wang, Teng Sun, Na Li, Yin Wang, Jian-Xun Wang, Lu-Yu Zhou, Bo Long, Cui-Yun Liu, Fang Liu, Pei-Feng Li

**Affiliations:** Division of Cardiovascular Research, State Key Laboratory of Biomembrane and Membrane Biotechnology, Institute of Zoology, Chinese Academy of Sciences, Beijing, China; The University of North Carolina at Chapel Hill, United States of America

## Abstract

Long noncoding RNAs (lncRNAs) are emerging as new players in gene regulation, but whether lncRNAs operate in the processing of miRNA primary transcript is unclear. Also, whether lncRNAs are involved in the regulation of the mitochondrial network remains to be elucidated. Here, we report that a long noncoding RNA, named mitochondrial dynamic related lncRNA (MDRL), affects the processing of miR-484 primary transcript in nucleus and regulates the mitochondrial network by targeting miR-361 and miR-484. The results showed that miR-361 that predominantly located in nucleus can directly bind to primary transcript of miR-484 (pri-miR-484) and prevent its processing by Drosha into pre-miR-484. miR-361 is able to regulate mitochondrial fission and apoptosis by regulating miR-484 levels. In exploring the underlying molecular mechanism by which miR-361 is regulated, we identified MDRL and demonstrated that it could directly bind to miR-361 and downregulate its expression levels, which promotes the processing of pri-miR-484. MDRL inhibits mitochondrial fission and apoptosis by downregulating miR-361, which in turn relieves inhibition of miR-484 processing by miR-361. Our present study reveals a novel regulating model of mitochondrial fission program which is composed of MDRL, miR-361 and miR-484. Our work not only expands the function of the lncRNA pathway in gene regulation but also establishes a new mechanism for controlling miRNA expression.

## Author Summary

Long non-coding RNAs (lncRNAs) have been shown to be involved in a wide range of biological functions. However, studies linking individual lncRNA to the mitochondrial fission program remain scarce. Also, it remains unknown whether lncRNAs can operate in the processing of miRNA primary transcript. Here, we provide causal evidence for the involvement of the lncRNA MDRL in the mitochondrial dynamics and the processing of miR-484 primary transcript in cardiomyocyte. We identified MDRL which can act as an endogenous ‘sponge’ that directly binds to miR-361 and downregulates its expression levels. miR-361 can directly bind to primary transcript of miR-484 and prevent its processing by Drosha into pre-miR-484. MDRL inhibits mitochondrial fission and apoptosis by miR-361 and miR-484. Our present study reveals a novel regulating model which is composed of MDRL, miR-361 and miR-484. Modulation of their levels may provide a new approach for tackling myocardial infarction.

## Introduction

Long non-coding RNAs (lncRNAs) are non-protein coding transcripts longer than 200 nucleotides. A number of lncRNAs have been shown to be involved in a wide range of biological functions including RNA processing [1], gene transcription regulation [2], miRNAs' host genes [3], modulation of apoptosis and invasion [4], marker of cell fate [5], chromatin modification [6], etc. Also, misregulation of lncRNAs has been observed in human diseases such as cancer and neurological disorders. In addition, lncRNAs not only can act as antisense transcripts or as decoy for splicing factors leading to splicing malfunctioning [7], [8], but also can act as a competing endogenous RNA (ceRNA) in mouse and human myoblasts [9]. However, it is not yet clear whether lncRNA can be involved in the processing of pri-miRNA and the regulation of mitochondrial network.

MicroRNAs (miRNAs) act as negative regulators of gene expression by inhibiting the translation or promoting the degradation of target mRNAs. Growing evidence has demonstrated that miRNAs can play a significant role in the regulation of development, differentiation, proliferation and apoptosis. Recent studies have identified the functional role of miRNA in numerous facets of cardiac biology, including the control of myocyte growth, contractility, fibrosis, angiogenesis, heart failure, and myocardial infarction, providing potential therapeutic targets for heart disease. To prevent and reverse myocardial infarction, it is critical to identify those miRNAs that are able to regulate myocardial infarction and to characterize their signal transduction pathways in the apoptotic cascades.

Mature miRNAs execute their functions mainly in the cytoplasm. Some studies have observed that there exist mature miRNAs in the nucleus [10], [11]. Their work demonstrated that additional sequence elements of specific miRNAs can control their posttranscriptional behavior, including the subcellular localization. Recent work reported that the miRNA pathway targets non-coding RNAs across species. It showed that let-7 could regulate its biogenesis autonomously through a conserved complementary site in its own primary transcript, creating a positive-feedback loop [11]. However, the molecular mechanism of miRNAs and their regulation model in the nucleus remains to be fully elucidated.

Mitochondria are highly dynamic organelles that constantly undergo fusion and fission to form a network that spans the entire area of the cell. Mitochondrial fission and fusion are crucial for maintaining mitochondrial function and are necessary for the maintenance of organelle fidelity [12]. The disruption of mitochondrial fission and fusion has been linked to the development and progression of some diseases [13]–[17]. Most recent studies have revealed that abnormal mitochondrial fusion and fission participate in the regulation of apoptosis. Mitochondrial fusion is able to inhibit apoptosis, while mitochondrial fission is necessary for initiation of apoptosis [12], [18]–[23]. Thus, exploring the function of mitochondrial fission and fusion regulators will unveil their roles in various pathway and diseases.

Our present work revealed that nuclear miR-361 can directly bind to pri-miR-484 and inhibiting its processing into pre-miR-484 which is mediated by Drosha. miR-361 participates in the regulation of mitochondrial network and apoptosis through the miR-484 pathway. Moreover, our study further suggested that a long noncoding RNA, MDRL, can directly bind to miR-361 and act as an endogenous “sponge” which downregulates miR-361 expression levels and promotes the processing of pri-miR-484. In short, MDRL regulates mitochondrial network and apoptosis through the miR-361 and miR-484. Our data reveal a novel role for lncRNA and miRNA in promoting biogenesis of other miRNA primary transcript, expanding the functions of the lncRNA and miRNA in gene regulation and mitochondrial network.

## Results

### miR-361 in the nucleus is able to regulate mature miR-484 levels

**Figure 1:**
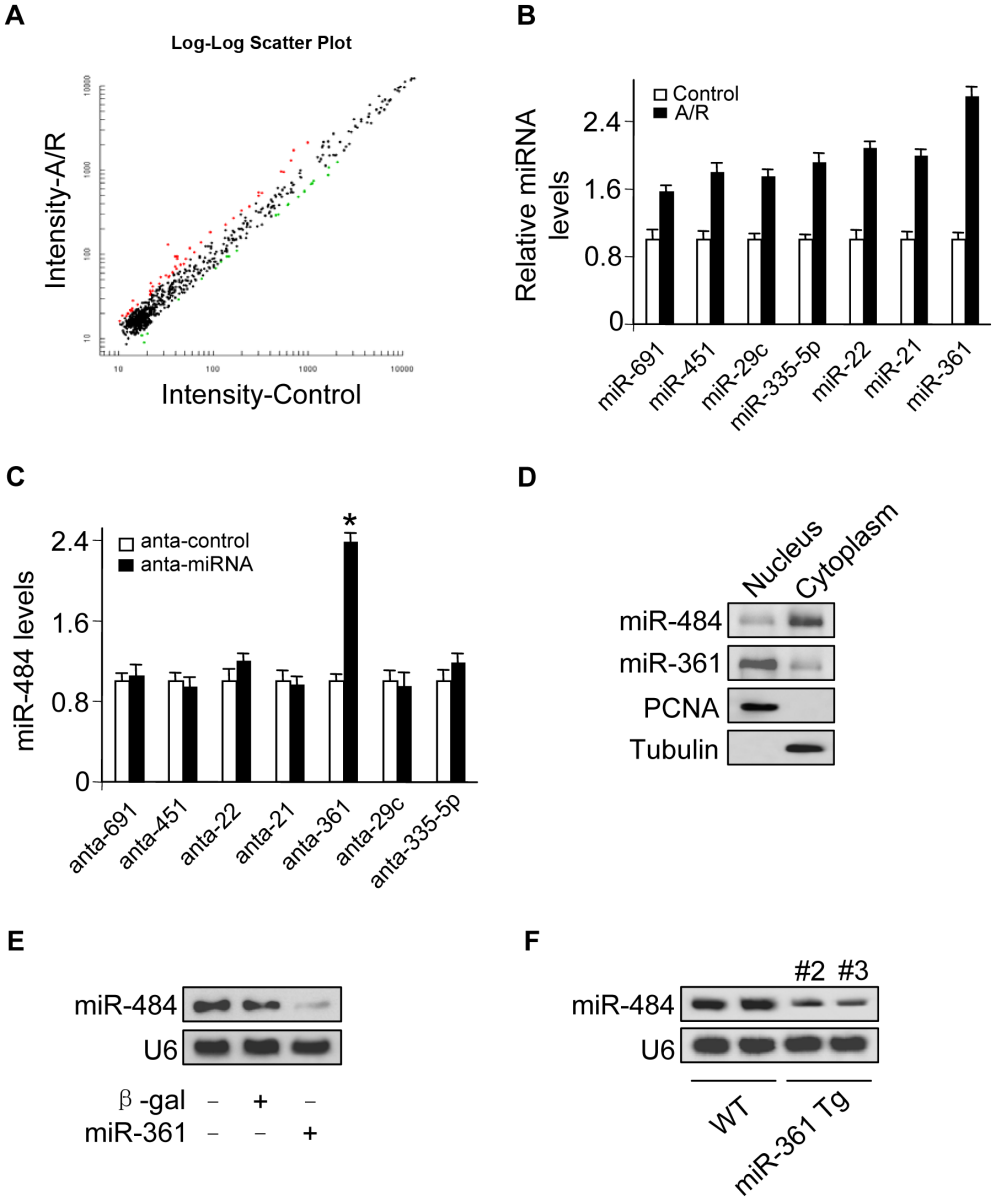
miR-361 in the nucleus is able to regulate mature miR-484 levels. **A.** Microarray results depicting the log-log scatter plot of intensity of miRNA expression in nuclei from control versus A/R treatment. The neonatal mouse cardiomyocytes were untreated (control) or exposed to A/R. The nuclei were purified and nuclear miRNAs were detected by microarray. The red dots and the green dots indicate 2 fold up- or down-regulated genes, respectively. **B.** Upregulated nuclear miRNAs upon A/R treatment. **C.** Knockdown of miR-361 elevates the levels of miR-484. Cardiomyocytes were transfected with indicated miRNA antagomir (anta-miRNA) or the antagomir control (anta-control). 48 h after transfection, miR-484 levels were analyzed by qRT-PCR. *p<0.05 vs anta-control. **D.** miR-361 predominantly localizes in the cell nucleus and miR-484 predominantly localizes in the cytoplasm. Total RNA was extracted from cardiomyocytes nucleus and cytoplasm. miR-361 and miR-484 levels were analyzed by northern blot. **E.** Enforced expression of miR-361 reduces the levels of miR-484. Cardiomyocytes were infected with adenoviral miR-361 or β-gal at a moi of 80. 24 h after infection, the expression of miR-484 was analyzed by northern blot. **F.** miR-361 suppresses the expression of miR-484 in the animal model. miR-484 levels in the hearts of WT and miR-361 transgenic mice were analyzed by northern blot.

Many studies have observed that there exist mature miRNAs in the nucleus [12], [18]–[23] and recent work also reports that let-7 miRNA in the nucleus can regulate its own primary transcript through a conserved complementary site, thus creating a positive-feedback loop [11]. Our previous work has showed that transcription factor could regulate miR-484 expression [24]. To further explore other underlying mechanism responsible for miR-484 regulation under anoxia/reoxygenation (A/R) condition, we tested whether miRNA in the nucleus participates in the regulation of miR-484 expression. To understand which nuclear miRNA is involved in the apoptosis pathway of A/R, we performed a microarray to detect nuclear miRNAs in response to A/R treatment (Figure 1A, Figure 1B and Table S1) and among these miRNAs induced by A/R, only knockdown of endogenous miR-361 (Figure S1A) induced an increase in the miR-484 expression levels (Figure 1C). A further study confirmed that miR-361 was predominantly located in the nucleus, and miR-484 was predominantly located in the cytoplasm (Figure 1D). We test whether nuclear miR-361 may directly affect the expression of miR-484. The results showed that enforced expression of miR-361 could reduce mature miR-484 levels (Figure 1E). Furthermore, miR-361 transgenic mice (Figures S1B and S1C) demonstrated reduced levels of miR-484 in the animal model (Figure S1D and Figure 1F). Taken together, it appears that miR-361 is predominantly located in the nucleus and is able to regulate mature miR-484 levels in the cellular and the animal model.

### miR-361 is able to directly bind to pri-miR-484 and inhibit its processing in the nucleus

**Figure 2:**
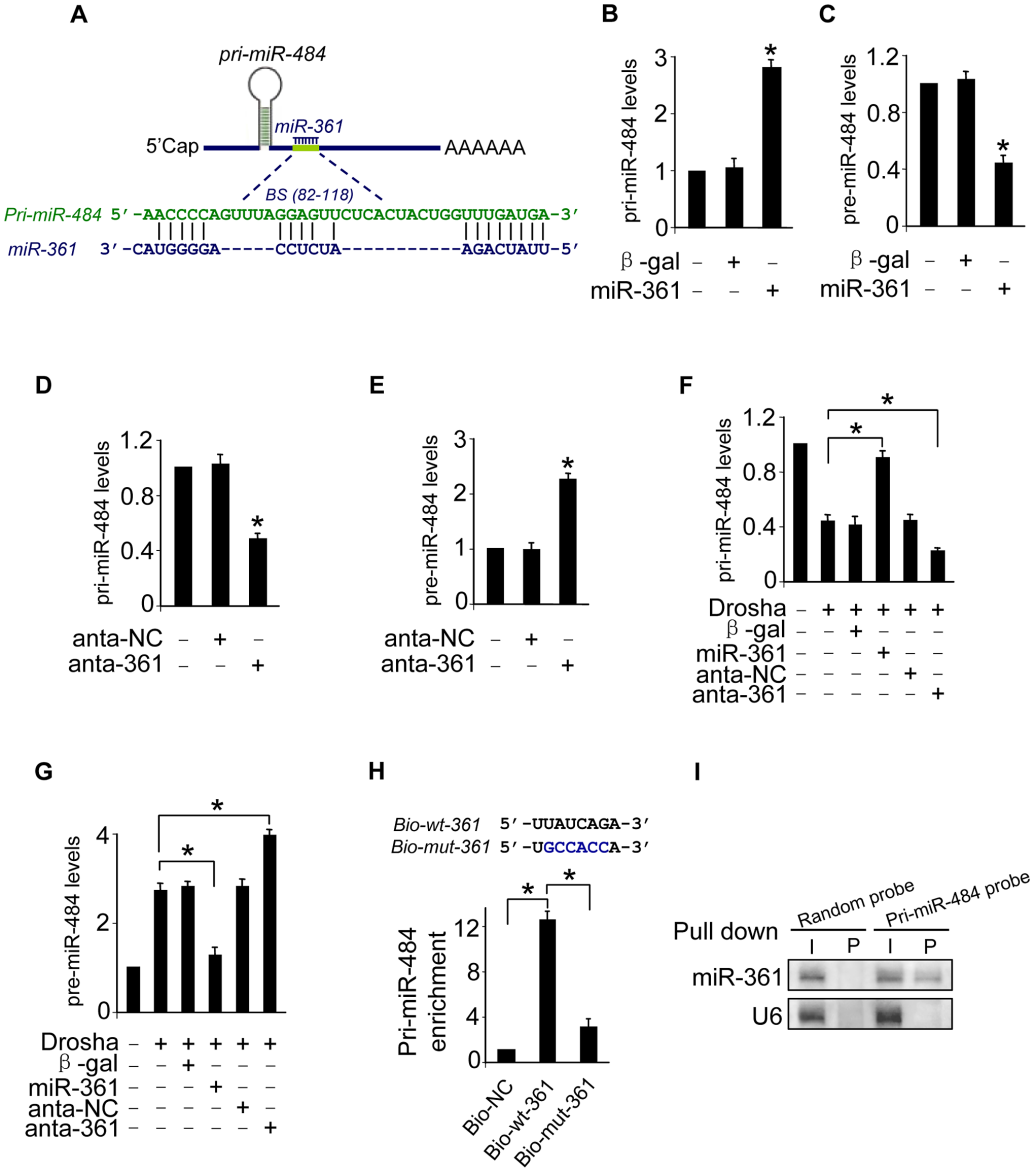
miR-361 can directly bind to pri-miR-484 and prevents its processing by Drosha into pre-miR-484. **A.** The miR-361 targeting site in pri-miR-484 is shown. **B** and **C.** Enforced expression of miR-361 increases the levels of pri-miR-484 and reduces the levels of pre-miR-484. Cardiomyocytes were infected with adenoviral miR-361 or β-gal at indicated time, the expression of pri-miR-484 (B) and pre-miR-484 (C) were analyzed by qRT-PCR. *p<0.05 vs control. **D** and **E.** Knockdown of miR-361 induces a reduction in pri-miR-484 levels and an increase in pre-miR-484. Cardiomyocytes were transfected with miR-361 antagomir (anta-361) or the antagomir negative control (anta-NC). 24 h after transfection, the expression of pri-miR-484 (D) and pre-miR-484 (E) were analyzed by qRT-PCR. *p<0.05 vs control. **F** and **G.** miR-361 prevents the processing of pri-miR-484 by Drosha into pre-miR-484. Cardiomyocytes were coinfected with the adenoviral Drosha and miR-361, transfected with anta-361 or anta-NC. 48 h after transfection, cells were harvested. pri-miR-484 (F) and pre-miR-484 (G) were analyzed by qRT-PCR. *p<0.05. **H.** miR-361 can directly bind to pri-miR-484 in vivo. Cardiomyocytes were transfected with biotinylated wild type miR-361 (Bio-wt-361), biotinylated mutant miR-361 (Bio-mut-361) and biotinylated negative control (Bio-NC). 48 h after transfection, cells were harvested for biotin-based pull-down assay. Only “seed” region of wt and mutant miR-361 were shown (upper panel). Pri-miR-484 were analyzed by qRT-PCR (low panel). *p<0.05. **I.** miR-361 is associated with pri-miR-484 in vivo. pri-miR-484 probe-coated magnetic bead was incubated with cardiomyocyte nulear lysate. After washing and enrichment of beads/RNA complex, RNA was eluted from the streptavidin beads and was analyzed by northern blot. I, input (10% samples were loaded); P, pellet (100% samples were loaded).

To understand the mechanism by which nuclear-located miR-361 regulates the levels of cytoplasmic mature miR-484, we tested whether miR-361 is able to affect the levels of pri-miR-484 located in nucleus. We compared the sequences of miR-361 with that of pri-miR-484 using the bioinformatics program RNAhybrid and noticed that miR-361 is complementary to pri-miR-484 (Figure 2A). Their complementary sequences led us to consider whether miR-361 can directly interact with pri-miR-484 and inhibit its processing into pre-miR-484 in the nucleus. We demonstrated that enforced expression of miR-361 resulted in the strong accumulation of pri-miR-484 (Figure 2B) and the reduction of pre-miR-484 (Figure 2C). Knockdown of miR-361 resulted in the reduction of pri-miR-484 (Figure 2D) and the increase of pre-miR-484 (Figure 2E). Thus, it appears that miR-361 prevents the processing of pri-miR-484 into pre-miR-484 in nucleus.

Mature miRNAs are generated via a two-step processing by Drosha and Dicer. The initial processing that occurs in the nucleus is catalyzed by Drosha. The Drosha complex cleaves pri-miRNA into pre-miRNA. To further verify whether miR-361 prevents the processing of Drosha. We applied Drosha to assay the levels of pri-miR-484 and pre-miR-484. Our data showed that enforced expression of miR-361 prevented the reduction of pri-miR-484 and inhibited the increase of pre-miR-484 induced by Drosha, and knockdown of miR-361 had an opposite effects (Figures 2F and 2G). These data suggest that miR-361 prevents the processing of pri-miR-484 by Drosha into pre-miR-484 in nucleus. To further test whether the miR-361 recognition element on pri-miR-484 was responsible for miR-361 binding and inhibition of processing, we applied a biotin-avidin pull-down system to assess the direct binding of miR-361 to pri-miR-484. The cardiomyocytes were transfected with biotinylated miR-361, biotinylated mutant miR-361 and biotinylated negative control (NC) (Figure S1E). Then the cells were harvested for biotin-based pull-down assay. Pri-miR-484 was co-precipitated, and the levels of pri-miR-484 in the pull-down complexes was analyzed by the qRT-PCR (Figure 2H). As shown in Figure 2H, pri-miR-484 was significantly enriched in the miR-361 pull-down products as compared to the biotinylated mutant miR-361 group and negative control group, indicating that miR-361 can directly bind to pri-miR-484 in vivo. We also employed inverse pull-down assay to test whether pri-miR-484 could pull down miR-361, a biotin-labeled-specific pri-miR-484 probe was used. The results showed that pri-miR-484 (Figure S1F) and miR-361 could be co-precipitated (Figure 2I). Taken together, it appears that miR-361 is able to directly bind to pri-miR-484 and prevent the processing of pri-miR-484 by Drosha into pre-miR-484.

### miR-361 regulates mitochondrial fission and apoptosis through miR-484

**Figure 3:**
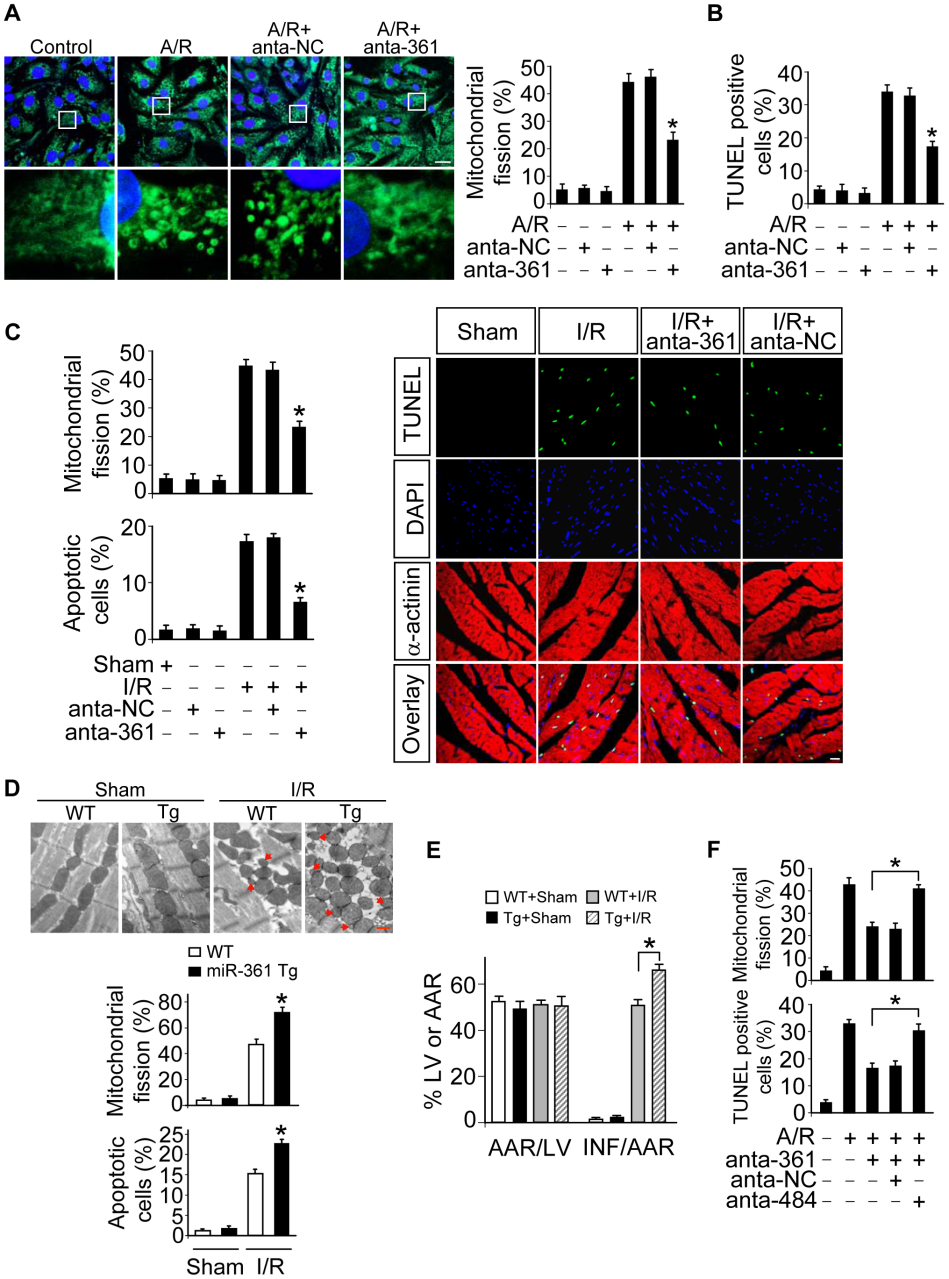
miR-361 provokes mitochondrial fission program. **A.** Knockdown of miR-361 prevents mitochondrial fission induced by A/R. Cardiomyocytes were transfected with miR-361 antagomir (anta-361) or the antagomir negative control (anta-NC), then exposed to A/R. The cells were stained with MitoTracker Green (left panel), Bar = 20 µm. The cells with fragmented mitochondria were counted (right panel). *p<0.05 versus A/R alone. **B.** Knockdown of miR-361 prevents apoptosis induced by A/R. Cardiomyocytes were treated as described for (A). Apoptosis was analyzed by TUNEL assay. *p<0.05 versus A/R alone. **C.** Knockdown of miR-361 attenuates mitochondrial fission upon I/R. Adult male C57BL/6 mice (8 weeks old) were delivered in three consecutive days, intravenous injections of miR-361 antagomir (anta-361) or antagomir control (anta-NC) at doses of 35 mg/kg body weight. 3 days after injection the mice were exposed to 45 min of ischemia and 3 hours of reperfusion. Counting of the fragmented mitochondria was shown in upper panel. Counting of the apoptotic cell was shown in low panel and right panel. TUNEL-positive myocyte nuclei (apoptotic cells) are green. Nuclei stained by DAPI show blue. Cardiomyocytes were labeled with α-actinin. *n* = 6, *p<0.05 versus I/R alone. **D** and **E.** miR-361 transgenic mice exhibited increased mitochondrial fission, apoptosis and myocardial infarction sizes in response to ischemia/reperfusion (I/R). Wild type C57BL/6 mice and miR-361 transgenic mice (8 weeks old) were exposed to 45 min of ischemia and 3 hours of reperfusion. Mitochondrial fission, apoptosis (D) and myocardial infarction sizes (E) were analyzed. *n* = 8, *p<0.05 versus WT+I/R. **F.** The knockdown of miR-484 attenuates the inhibitory effect of miR-361 knockdown on mitochondria fission and apoptosis. Cardiomyocytes were transfeted with the miR-361 antagomir, miR-484 antagomir or antagomir negative control, and then treated with A/R. Mitochondria fission and apoptosis were analyzed. *p<0.05.

Our previous findings found that miR-484 could inhibit mitochondrial fission and apoptosis in cardiomyocytes [24]. The present result appears that miR-361 can interact with pri-miR-484 and regulate mature miR-484 levels. We thus explored the functional role of miR-361 in mitochondrial fission and apoptosis. To this end, the antagomir of miR-361 was employed to knock down endogenous miR-361. Mitochondrial fission induced by A/R was attenuated by the knockdown of miR-361 (Figure 3A). Concomitantly, apoptosis was reduced in the presence of miR-361 antagomir (Figure 3B). These data indicate that miR-361 can promote mitochondrial fission and apoptosis upon A/R treatment.

To understand the pathophysiological role of miR-361, we detected whether miR-361 is involved in the pathogenesis of myocardial infarction in the animal model. miR-361 was elevated in response to ischemia/reperfusion (Figure S2A). Knockdown of miR-361 resulted in a reduction in mitochondrial fission (Figure 3C, upper panel) and apoptosis (Figure 3C, low panel and right panel). We produced miR-361 transgenic mice, and these mice exhibited increased mitochondrial fission, apoptosis (Figure 3D), myocardial infarction sizes (Figure 3E) and potentiated cardiac dysfunction (Figure S2B) in response to ischemia/reperfusion (I/R). Taken together, it appears that miR-361 is able to promote mitochondrial fission, apoptosis and myocardial infarction. How does miR-361 exert its effect on the mitochondrial network? Because miR-361 is able to reduce mature miR-484 expression as shown in Figure 1, we thus tested whether miR-484 is a mediator of miR-361. To confirm the relationship between miR-361 and miR-484 in mitochondrial fission machinery, we used antagomir to inhibit miR-484 levels, and observed that the inhibitory effect of miR-361 knockdown on mitochondrial fission and apoptosis was attenuated in the presence of miR-484 antagomir (Figure S3A and Figure 3F). Taken together, these data suggest that miR-361 targets miR-484 in the cascades of mitochondrial fission and apoptosis.

### MDRL is able to regulate miR-361 expression and activity

**Figure 4:**
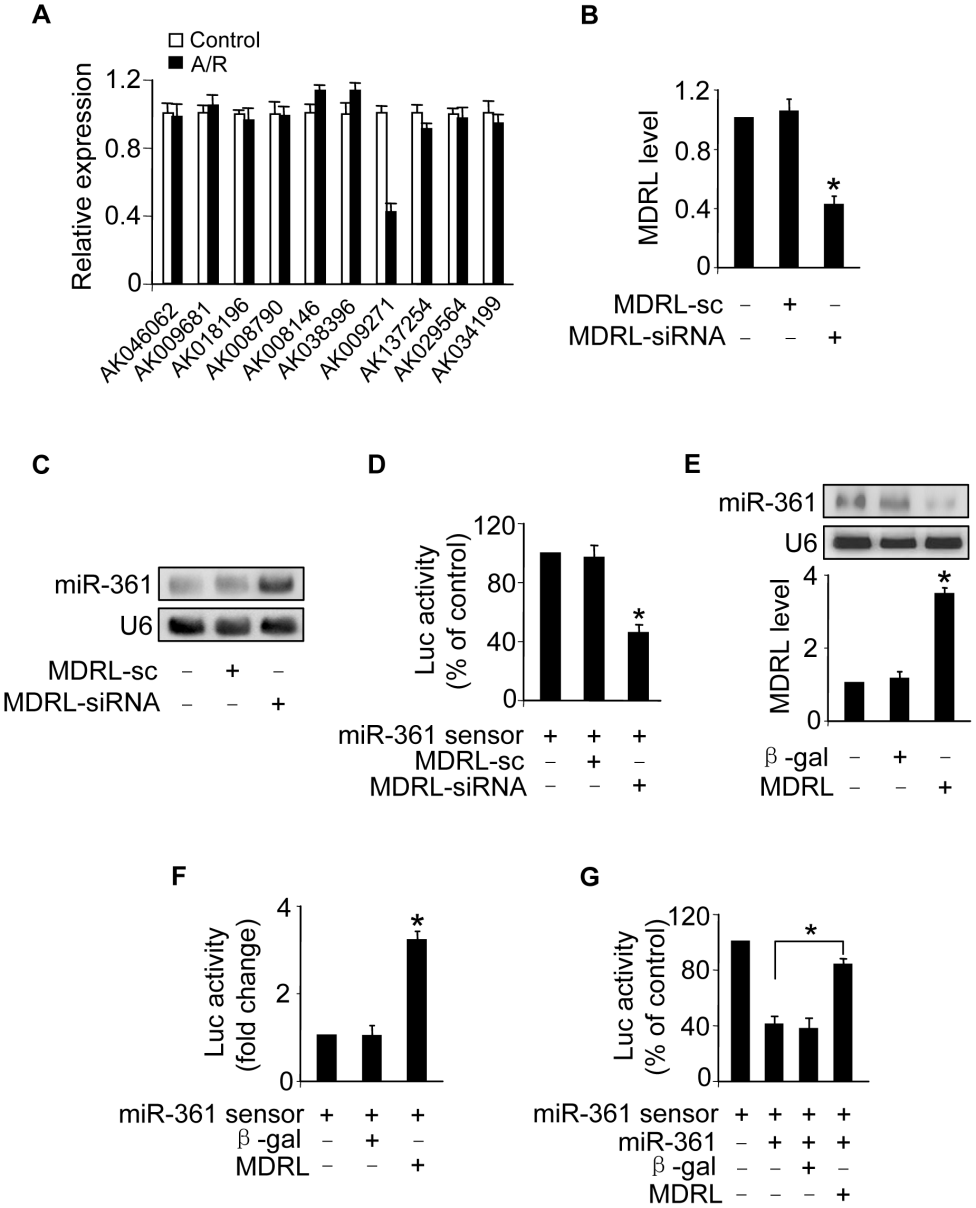
MDRL can regulate miR-361 expression and activity. **A.** LncRNAs expression levels upon treatment with A/R. Cardiomyocytes were untreated (control) or treated with A/R. LncRNAs expressed in heart in lncRNA array from Affymetrix company (http://www.noncode.org) were analyzed by qRT-PCR. *p<0.05 vs control. **B.** Knockdown of MDRL induces the decrease of MDRL expression levels. Cardiomyocytes were infected with adenoviral MDRL siRNA (MDRL-siRNA) and its scramble form (MDRL-sc). 24 h after infection MDRL levels were analyzed by real time PCR. *p<0.05 vs control. **C.** Knockdown of MDRL upregulates miR-361 expression levels. Cardiomyocytes were infected with adenoviral MDRL siRNA (MDRL-siRNA) and its scramble form (MDRL-sc). 24 h after infection miR-361 levels were analyzed by northern blot. **D.** Knockdown of MDRL induces miR-361 activity. Cardiomyocytes were infected with adenoviral MDRL-siRNA and its scramble, then transfected with miR-361 sensor. Luciferase activity was analyzed. *p<0.05 vs miR-361 sensor alone. **E.** Enforced expression of MDRL reduces the expression levels of miR-361. Cardiomyocytes were infected with adenoviral MDRL or β-gal. 24 h after infection MDRL levels were analyzed by real time PCR (low panel), and miR-361 levels were analyzed by northern blot (upper panel). **F.** MDRL reduces miR-361 activity. Cardiomyocytes were infected with adenoviral MDRL or β-gal, then transfected with miR-361 sensor. Luciferase activity was analyzed. *p<0.05 vs miR-361 sensor alone. **G.** MDRL acts as a sponge for miR-361 activity. Cardiomyocytes were infected with adenoviral miR-361, MDRL or β-gal, then transfected with miR-361 sensor. Luciferase activity was analyzed. *p<0.05.

Recent studies have suggested that lncRNAs may act as endogenous sponge RNA to interact with miRNAs and influence the expression of miRNA [9], [25]–[27]. To explore the underlying mechanism responsible for miR-361 upregulation in response to A/R treatment, we tested whether lncRNA could regulate miR-361 expression. We carried out qRT-PCR to detect lncRNAs levels in response to A/R treatment. LncRNAs were chosen from the lncRNA array published online by Fantom company. Among 100 lncRNAs, AK009271 which we named mitochondrial dynamic related lncRNA (MDRL), was substantially reduced (Figure 4A). The MDRL is 1039 nt in length and the subcellular location showed that MDRL was expressed both in nucleus and cytoplasm (Figure S3B). Further, our results showed that miR-361 levels were elevated in the cells upon knockdown of endogenous MDRL (Figures 4B and 4C). To know whether MDRL can affect miR-361 activity, we constructed miR-361 sensor (with a perfect miR-361 binding site). The lucifease activity of miR-361 sensor was decreased in cells treated with MDRL siRNA (Figure 4D), suggesting the induction of miR-361 activity. Enforced expression of MDRL induced a reduction in miR-361 expression (Figure 4E) and activity (Figure 4F). To further test whether MDRL may act as a miR-361 sponge, we transfected the miR-361 sensor luciferase reporter, along with adenoviral miR-361, MDRL or β-gal. The luciferase activity showed that MDRL counteracted the effect of miR-361 (Figure 4G), suggesting that MDRL is a functional sponge for miR-361. Taken together, these data suggest that MDRL is able to regulate miR-361 levels and activity.

### MDRL is able to directly bind to miR-361 in vivo

**Figure 5:**
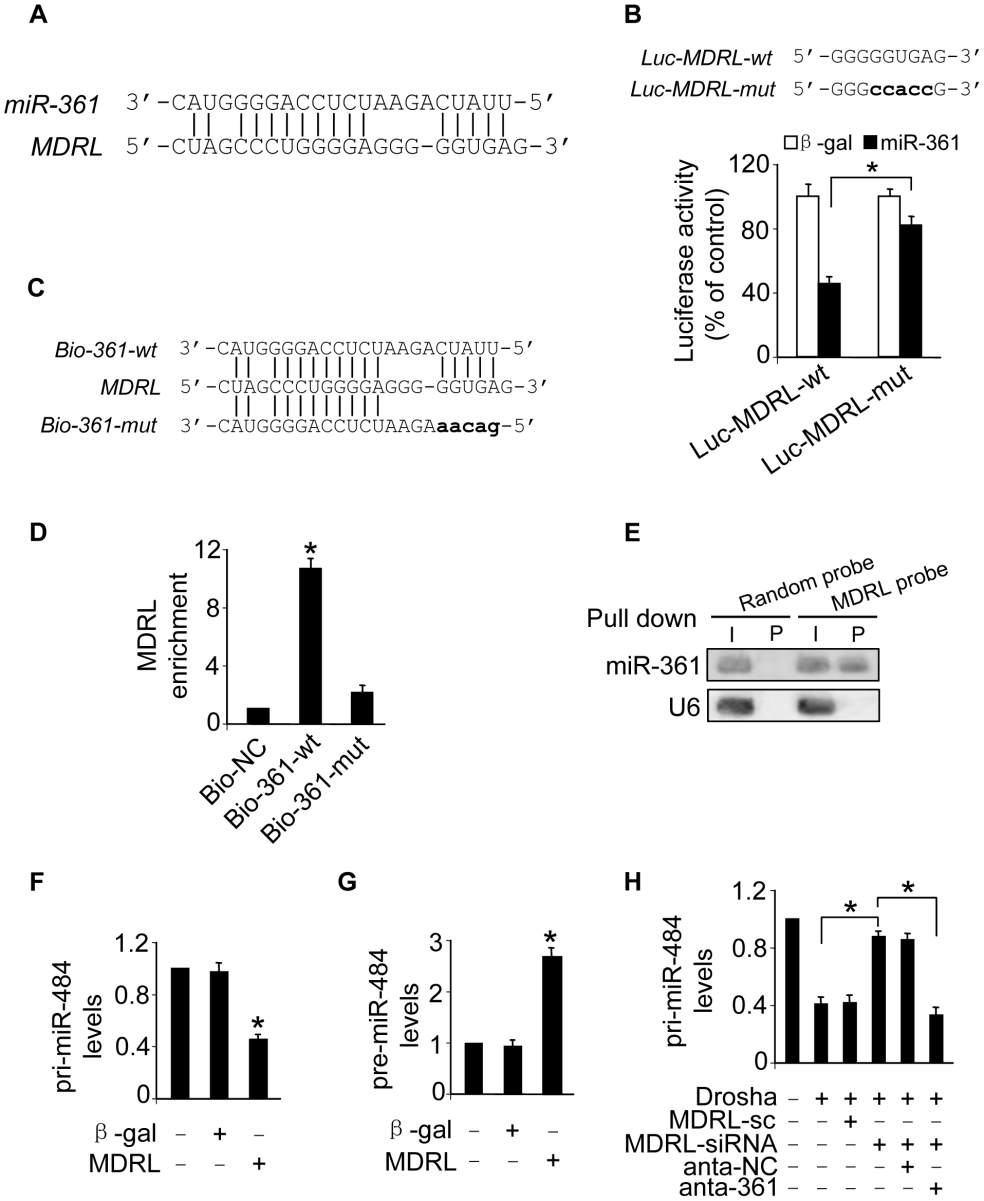
Interaction between MDRL and miR-361. **A.** MDRL RNA contains a site complementary to miR-361. **B.** Luciferase assay. miR-361 binding site in MDRL RNA wild type form (Luc-MDRL-wt) and the mutated form (Luc-MDRL-mut) are shown in upper panel. HEK293 cells were infected with adenoviral miR-361 or β-gal, then transfected with the luciferase constructs of Luc-MDRL-wt or Luc-MDRL-mut. The luciferase activity was analyzed. *p<0.05. **C.** Wild type and the mutated form of biotin-labeled miR-361 sequence are shown. **D.** miR-361 can bind directly to MDRL in vivo. Cardiomyocytes were transfected with biotinylated wild type miR-361 (Bio-wt-361) or biotinylated mutant miR-361 (Bio-mut-361). A biotinylated miRNA that is not complementary to MDRL was used as a negative control (Bio-NC). 48 h after transfection, cells were harvested for biotin-based pull-down assay. MDRL expression levels were analyzed by real time PCR. *p<0.05 vs Bio-NC. **E.** MDRL can bind to miR-361 in vivo. Cardiomyocyte nuclear lysate was incubated with MDRL probe or random probe-coated magnetic bead. After washing and enrichment of beads/RNA complex, RNA was eluted from the streptavidin beads and was analyzed by northern blot. I, input (10% samples were loaded); P, pellet (100% samples were loaded). **F** and **G.** Enforced expression of MDRL induces the decreases of pri-miR-484 expression levels and the increases pre-miR-484 expression levels. Cardiomyocytes were infected with adenoviral MDRL or β-gal, the expression of pri-miR-484 (F) and pre-miR-484 (G) were analyzed by qRT-PCR. *p<0.05 vs control. **H.** Knockdown of miR-361 attenuated the inhibitory effects of MDRL knockdown on the processing of pri-miR-484 induced by Drosha. Cardiomyocytes were coinfected with the adenoviral Drosha, MDRL-siRNA and MDRL-sc, transfected with anta-361 or anta-NC. 48 h after transfection, cells were harvested. pri-miR-484 expression levels were analyzed by qRT-PCR. *p<0.05.

To understand the mechanism by which MDRL regulates the levels of miR-361, we tested whether MDRL can interact with miR-361. We compared the sequences of MDRL with that of miR-361 using the bioinformatics program RNAhybrid and noticed that MDRL contains a target site of miR-361 (Figure 5A). The wild type luciferase construct of MDRL (Luc-MDRL-wt) and a mutated form (Luc-MDRL-mut) were produced by inserting the sequence of putative miR-361 binding site into the report constructs (Figure 5B, upper panel). Luciferase assay revealed that miR-361 could suppress the luciferase activity of MDRL, but it had less effect on the mutated form of MDRL compared to the wild type (Figure 5B). Our results further showed that the mutated form of MDRL had no effect on miR-361 activity (Figure S3C) and it also lost the ability to counteract miR-361 (Figure S3D). These results revealed that MDRL may interact with miR-361 by this putative binding site.

Further, we applied a biotin-avidin pull-down system to test if miR-361 could pull down MDRL. Cardiomyocytes were transfected with biotinylated wild type miR-361, biotinylated mutant miR-361 and biotinylated miR-NC (Figure S4A). We found these transfections did not change MDRL levels (Figure S4B). Then, Cardiomyocytes were harvested for biotin-based pull-down assay. MDRL was pulled down by wild type miR-361 as analyzed by qRT-PCR, but the introduction of mutations that disrupt base pairing between MDRL and miR-361 (Figure 5C) led to the inability of miR-361 to pull down MDRL (Figure 5D), indicating that the recognition of miR-361 to MDRL is in a sequence-specific manner. We also employed inverse pull-down assay to test if MDRL could pull down miR-361, a biotin-labeled-specific MDRL probe was used. The results showed that MDRL (Figure S4C) and miR-361 (Figure 5E) could be co-precipitated. Taken together, it appears that MDRL is able to directly bind to miR-361 in vivo.

We further tested whether MDRL is able to regulate miR-484 expression and influence its processing. Our results showed that enforced expression of MDRL (Figure S4D) resulted in the decrease of pri-miR-484 (Figure 5F) and the accumulation of pre-miR-484 (Figure 5G). Knockdown of MDRL induced the increase of pri-miR-484 (Figure S4E) and the decrease of pre-miR-484 (Figure S4F). Knockdown of MDRL inhibited the decrease of pri-miR-484 (Figure 5H) and the increase of pre-miR-484 (Figure S4G) induced by Drosha, and the inhibitory effect of MDRL knockdown was reduced in the presence of miR-361 antagomir (Figure 5H and Figure S4G). Thus, it appears that MDRL promotes the processing of pri-miR-484 into pre-miR-484 through targeting miR-361.

### MDRL regulates mitochondrial fission and apoptosis through miR-361 and miR-484

**Figure 6:**
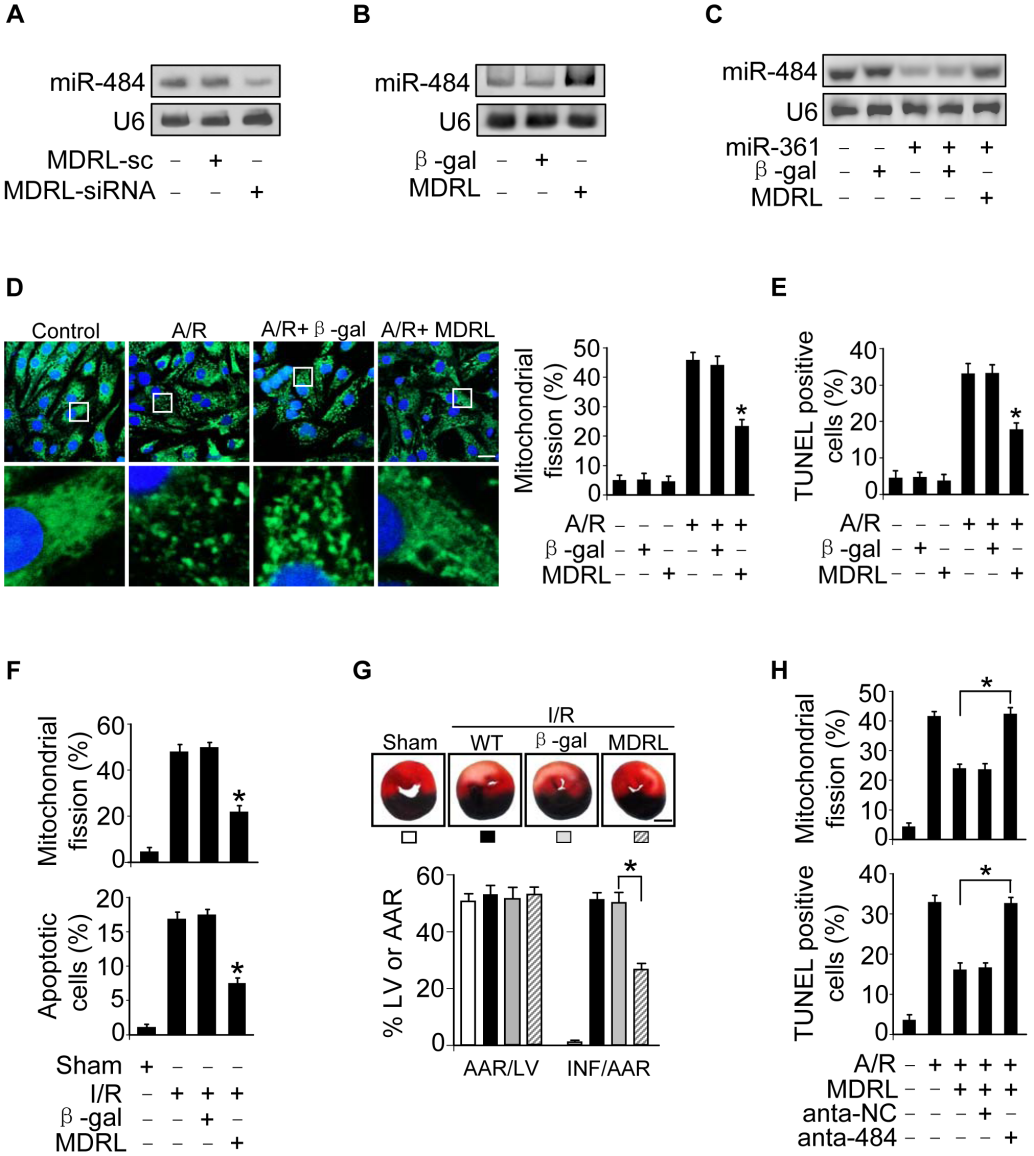
MDRL regulates mitochondrial fission and apoptosis through targeting miR-361 and miR-484. **A.** Knockdown of MDRL reduces the expression levels of miR-484. Cardiomyocytes were infected with adenoviral MDRL-siRNA or MDRL-sc. 24 h after infection miR-484 levels were analyzed by northern blot. **B.** Enforced expression of MDRL induces the increase of miR-484 levels. Cardiomyocytes were infected with adenoviral MDRL or β-gal. 24 h after infection miR-484 levels were analyzed by northern blot. **C.** MDRL reduces the inhibitory effect of miR-361 on miR-484 expression. Cardiomyocytes were infected with adenoviral miR-361, MDRL or β-gal. miR-484 expression levels were analyzed by northern blot. **D.** MDRL inhibits mitochondrial fission induced by A/R. Cardiomyocytes were infected with adenoviral MDRL or β-gal, and were exposed to A/R. The cells were stained with MitoTracker Green (left panel), Bar = 20 µm. The cells with fragmented mitochondria were counted (right panel). *p<0.05 vs A/R alone. **E.** MDRL inhibits apoptosis induced by A/R. Cardiomyocytes were infected with adenoviral MDRL or β-gal, then were exposed to A/R. Apoptosis was assessed by TUNEL assay. *p<0.05 vs A/R alone. **F.** MDRL could inhibit mitochondrial fission and apoptosis upon I/R. Intracoronary delivery of adenoviral constructs of MDRL or β-gal to the hearts was described in the section of Materials and Methods. Mice were subjected to 45 min ischemia and 3 h reperfusion. Counting of fragmented mitochondria and apoptosis were shown. *p<0.05 versus I/R alone. **G.** MDRL attenuates myocardial infarction upon I/R. Mice were treated as described in (F). The infarct sizes were shown. *p<0.05. Bar = 2 mm. **H.** Knockdown of miR-484 attenuates the inhibitory effect of MDRL on mitochondria fission and apoptosis. Cardiomyocytes were infected with the adenoviral MDRL, miR-484 antagomir or antagomir-NC, and then treated with A/R. Mitochondria fission and apoptosis were analyzed. *p<0.05.

Our present results have demonstrated that MDRL could promote the processing of pri-miR-484 by Drosha. Thus, we tested whether MDRL is able to regulate mature miR-484 levels. Knockdown of MDRL reduced miR-484 levels (Figure 6A), while overexpression of MDRL resulted in up-regulation of miR-484 expression (Figure 6B). And MDRL counteracted the effect of miR-361 on miR-484 expression (Figure 6C). Our previous report has demonstrated that Fis1 is a downstream target of miR-484. The current data showed that MDRL could regulate Fis1 expression by miR-484 (Figure S5A). These results indicated that MDRL may act as endogenous sponge “antagomir” of miR-361 to regulate the processing of pri-miR-484 and expression of miR-484.

To explore the functional role of MDRL, we tested that enforced expression of MDRL inhibited mitochondrial fission (Figure 6D) and apoptosis (Figure 6E and Figure S5B) induced by A/R. We also demonstrated that knockdown of MDRL induced mitochondrial fission (Figure S5C) and apoptosis (Figure S5D). In the animal model, administration of MDRL attenuated mitochondrial fission, cell death (Figure 6F) and myocardial infarction sizes (Figure 6G) in response to ischemia/reperfusion (I/R). Administration of MDRL also ameliorated cardiac function (Figure S5E). Taken together, it appears that MDRL is able to prevent mitochondrial fission, apoptosis and myocardial infarction. Since MDRL is able to elevate miR-484 expression as shown in Figure 6B, we thus tested whether miR-484 is a mediator of MDRL in the mitochondrial network. To confirm the relationship between MDRL and miR-484 in mitochondrial fission machinery, we used miR-484 antagomir, and observed that the inhibitory effect of MDRL on mitochondrial fission and apoptosis was decreased in the presence of miR-484 antagomir (Figure 6H). Taken together, these data suggest that MDRL targets miR-361/miR-484 in the cascades of mitochondrial fission and apoptosis (Figure S6).

## Discussion

Our present work revealed that miR-361 directly binds to pri-miR-484 and prevents its processing by Drosha into pre-miR-484. miR-361 is able to regulate mitochondrial fission and apoptosis, and this regulatory effects on mitochondrial fission and apoptosis is through targeting miR-484. Our study further revealed that MDRL directly binds to miR-361 and acts as its “sponge”, promoting the processing of pri-miR-484. And MDRL can inhibit mitochondrial fission and apoptosis though targeting miR-361 and miR-484. Our results provide novel evidence demonstrating that MDRL, miR-361, miR-484 constitute an axis in the machinery of mitochondrial network.

LncRNAs have been defined to have important functions in specific cell types, tissues and developmental conditions such as chromatin modification [6], RNA processing [1], structural scaffolds [28] and modulation of apoptosis and invasion, etc [4]. Despite the biological importance of lncRNAs, it is not yet clear whether lncRNAs is involved in the processing of primary transcript and the regulation of mitochondrial network. Our present work for the first time reveals a novel function of lncRNA participating in regulating the processing of miR-484 primary transcript and mitochondrial dynamics. Our results may provide a new clue for the understanding of lncRNAs-controlled cellular events.

It has been shown that lncRNAs may act as endogenous sponge RNAs to interact with miRNAs and influence the expression of miRNA target genes. A recent report shows that the H19 lncRNA can act as a molecular sponge for the major let-7 family of miRNAs [27]. Other report demonstrates that a muscle-specific long non-coding RNA, linc-MD1, governs the time of muscle differentiation by acting as a competing endogenous RNA (ceRNA) in mouse and human myoblasts [9]. Highly up-regulated liver cancer (HULC) may act as an endogenous ‘sponge’, which down-regulates miR-372 leading to reducing translational repression of its target gene, PRKACB [26]. Transient knockdown and ectopic expression of HSUR 1 direct degradation of mature miR-27 in a sequence-specific and binding-dependent manner [25]. Our present study reveals that lncRNA (MDRL) sponges miR-361 and promoting the processing of pri-miR-484, which inhibits mitochondrial fission and apoptosis. The discovery of a long non-coding RNA in miRNA primary processing and mitochondrial dynamics may shed new lights on understanding the complex molecular mechanism of mitochondrial network.

Many research works reveal that mature miRNAs execute their functions mainly in the cytoplasm. Recently, it has been reported that miRNA also functions in nucleus [12], [18]–[23], but the function of nuclear miRNA remains to be fully unveiled. Mature miRNAs are generated via a two-step processing by Drosha and Dicer. The initial processing that occurs in the nucleus is catalyzed by Drosha. The Drosha complex cleaves pri-miRNA into pre-miRNA. The precise processing is pivotal to ensure the production of mature miRNA. This present work reveals that miR-361 in the nucleus can directly bind to the pri-miR-484 and prevent its processing by Drosha into pre-miR-484, and then further inhibit the biological function of miR-484. This finding may provide a new clue for the understanding of miRNAs-controlled gene expression.

Emerging data suggest that changes in mitochondrial morphology may be relevant to various aspects of cardiovascular biology including cardiac development, heart failure, diabetes mellitus, and apoptosis. The heart function stringently depends on the ATP-generating pathways [29], and cardiomyocytes are a good model to study mitochondrial dynamics because of the abundant existence of mitochondria. So far, it remains unclear whether lncRNA is involved in the regulation of mitochondrial dynamics. Our present work indicated that lncRNA (MDRL) can inhibit mitochondrial fission and apoptosis through regulating miR-361 and miR-484. The involvement of MDRL, miR-361 and miR-484 in regulating mitochondrial networks shed new lights on the understanding of mitochondrial integrity and cardiac pathophysiology.

In summary, our present study reveals that miR-361 located in nucleus can directly bind to pri-miR-484 and prevent its processing by Drosha into pre-miR-484. miR-361 reduces mature miR-484 levels and affects mitochondrial apoptotic pathway through targeting miR-484. Moreover, we demonstrated that MDRL acts as endogenous sponge RNA and inhibits miR-361 expression. MDRL is able to inhibit mitochondrial fission and apoptosis through targeting miR-361 and miR-484. Thus, modulation of MDRL and miR-361 may represent novel approaches for interventional treatment of cardiac disease. This finding may provide a new clue for the understanding of lncRNAs and miRNAs-controlled cellular events.

## Materials and Methods

### Ethics statement

We declare that all experiments were performed according to the protocols approved by the Animal Care Committee, Institute of Zoology, Chinese Academy of Sciences, China.

### Cardiomyocytes culture and treatment

Neonatal mouse cardiomyocytes were isolated and prepared as we described [30]. In brief, after dissection the hearts were washed, minced in HEPES-buffered saline solution containing 130 mM NaCl, 3 mM KCl, 1 mM NaH_2_PO_4_, 4 mM glucose and 20 mM HEPES (pH adjusted to 7.35 with NaOH). Tissues were then dispersed in a series of incubations at 37°C in HEPES-buffered saline solution containing 1.2 mg/ml pancreatin and 0.14 mg/ml collagenase (Worthington). After centrifugation the cells were re-suspended in Dulbecco's modified Eagle medium/F-12 (GIBCO) containing 5% heat-inactivated horse serum, 0.1 mM ascorbate, insulin-transferring-sodium selenite media supplement, 100 U/ml penicillin, 100 µg/ml streptomycin, and 0.1 mM bromodeoxyuridine. The dissociated cells were pre-plated at 37°C for 1 h. The cells were then diluted to 1×10^6^ cells/ml and plated in 10 µg/ml laminin-coated different culture dishes according to the specific experimental requirements. Anoxia/reoxygenation was performed as follows. Briefly, cells were placed in an anoxic chamber with a water-saturated atmosphere composed of 5% CO_2_ and 95% N_2_. Cells were subjected to 6 hours of anoxia followed by 12 hours of reoxygenation (95% O_2_ and 5% CO_2_).

### Generation of cardiac specific miR-361 transgenic mice

For creating miR-361 transgenic mice, a DNA fragment containing murine miR-361 was cloned to the vector, pαMHC-clone26 (kindly provided by Dr. Zhong Zhou Yang), under the control of the α-myosin heavy chain (α-MHC) promoter. The primers used to generate miR-361 transgenic mice include, forward primer: 5′-AGAATGAGGCTAACAGGTGAGTCATC-3′; reverse primer: 5′-TGACTGGCAGACACTGGTTTCAGGTGTTAC-3′. Microinjection was performed following standard protocols.

### Mitochondrial staining

Mitochondrial staining was carried as we and others described with modifications [19], [26]. Briefly, cells were plated onto the cover-slips coated with 0.01% poly-L-lysine. After treatment they were stained for 30 min with 0.02 µM MitoTracker Green (Molecular Probes). Mitochondria were imaged using a laser scanning confocal microscope (Zeiss LSM510 META).

### Statistical analysis

Data are expressed as the mean ± SEM of at least three independent experiments. We evaluated the data with Student's *t* test. We used a one-way analysis of variance for multiple comparisons. A value of p<0.05 was considered significant.

## Supporting Information

Figure S1 miR-361 transgenic mice genotyping assay. **A.** miRNAs levels are knocked down by transfecting cardiomyocytes with antagomir. Cardiomyocytes were transfected with indicated miRNA antagomir (anta-miRNA) or the antagomir control (anta-control). 48 h after transfection, the expression of miRNAs were analyzed by qRT-PCR. **B.** Schematic map showing the construct of miR-361 transgenic mice. **C.** The expression of miR-361 was analyzed by qRT-PCR from wild type and miR-361 transgenic mice of different lines. The results were normalized to U6. **D.** miR-361 levels analysis. miR-361 levels in the hearts of WT and miR-361 transgenic mice were analyzed by qRT-PCR. **E.** The transfection efficiency of miR-361. Cardiomyocytes were transfected with biotinylated wild type miR-361 (Bio-wt-361), biotinylated mutant miR-361 (Bio-mut-361) and biotinylated negative control (Bio-NC). The expression levels of miR-NC, miR-361 and miR-361 mut were analyzed by northern blot. **F.** pri-miR-484 levels assay. pri-miR-484 probe-coated magnetic bead was incubated with cardiomyocyte nulear lysate. After washing and enrichment of beads/RNA complex, RNA was eluted from the streptavidin beads and pri-miR-484 levels were analyzed by qRT-PCR. *p<0.05 vs Random probe.
(PDF)

Figure S2 miR-361 is upregulated upon ischemia/reperfusion. **A.** miR-361 levels during myocardial ischemia/reperfusion. Mice were induced to undergo cardiac ischemia/reperfusion. Area-at-risk and the remote area were prepared at the indicated time for qRT-PCR analysis of miR-361 levels. n = 7, *p<0.05 vs 0 min or sham. **B.** miR-361 transgenic mice exhibit more severe cardiac dysfunction upon I/R. Mice were treated as described above. Transthoracic echocardiographic analysis was performed at 1 week after sham or I/R. LVIDd, diastolic left ventricular internal diameters; LVIDs, systolic left ventricular internal diameters; FS, fractional shortening of left ventricular diameter. *n* = 8, *p<0.05.
(PDF)

Figure S3 MDRL regulates miR-361 activity. **A.** miR-484 levels analysis. Cardiomyocytes were transfeted with the miR-361 antagomir, miR-484 antagomir or antagomir negative control, and then treated with A/R. miR-484 levels were analyzed by qRT-PCR. *p<0.05. **B.** Detection of MDRL in nuclear or cytoplasmic fractions in cardiomyocytes. The levels of MDRL were analyzed by northern blot. **C.** MDRL reduces miR-361 activity. Cardiomyocytes were infected with adenoviral MDRL-wt, MDRL-mut or β-gal, then transfected with miR-361 sensor. Luciferase activity was analyzed. *p<0.05. **D.** MDRL acts as a sponge for miR-361 activity. Cardiomyocytes were infected with adenoviral miR-361, MDRL-wt, MDRL-mut or β-gal, then transfected with miR-361 sensor. Luciferase activity was analyzed. *p<0.05.
(PDF)

Figure S4 MDRL regulates the processing of pri-miR-484. **A.** The transfection efficiency of miR-361. Cardiomyocytes were transfected with biotinylated wild type miR-361 (Bio-361-wt), biotinylated mutant miR-361 (Bio-361-mut) and biotinylated negative control (Bio-NC). The expression levels of miR-NC, miR-361 and miR-361 mut were analyzed by northern blot. **B.** Cardiomyocytes were treated as described above. The expression levels of MDRL were analyzed by qRT-PCR. **C.** Cardiomyocyte nuclear lysate was incubated with MDRL probe or random probe-coated magnetic bead. After washing and enrichment of beads/RNA complex, RNA was eluted from the streptavidin beads and MDRL was analyzed by qRT-PCR. *p<0.05 vs Random probe. **D.** The expression levels of MDRL. Cardiomyocytes were infected with adenoviral MDRL or β-gal. MDRL levels were analyzed by qRT-PCR. *p<0.05 vs control. **E** and **F.** Knockdown of MDRL led to the accumulation of pri-miR-484 and the reduction of pre-miR-484 levels. Cardiomyocytes were infected with adenoviral MDRL-siRNA or MDRL-sc, the expression of pri-miR-484 (E) and pre-miR-484 (F) were analyzed by qRT-PCR. *p<0.05 vs control. **G.** Knockdown of miR-361 attenuated the inhibitory effects of MDRL knockdown on the processing of pri-miR-484 induced by Drosha. Cardiomyocytes were coinfected with the adenoviral Drosha, MDRL-siRNA and MDRL-sc, transfected with anta-361 or anta-NC. 48 h after transfection, cells were harvested. pre-miR-484 expression levels were analyzed by qRT-PCR. *p<0.05.
(PDF)

Figure S5 Knockdown of MDRL induces mitochondrial fission and apoptosis. **A.** MDRL regulates Fis1 expression by miR-484. Cardiomyocytes were infected with the adenoviral MDRL, miR-484 antagomir or antagomir-NC. The expression levels of Fis1 were analyzed by immunoblot. **B.** MDRL inhibits apoptosis induced by A/R. Cardiomyocytes were infected with adenoviral MDRL or β-gal, and then were exposed to A/R. TUNEL was employed to analyze apoptotic cells. Bar = 50 µm. **C** and **D.** Knockdown of MDRL promotes mitochondrial fission and apoptosis. Cardiomyocytes were infected with adenoviral MDRL-siRNA or MDRL-sc. Mitochondrial fission (C) and apoptosis (D) were analyzed. **E.** Intracoronary delivery of adenoviral constructs of MDRL or β-gal to the hearts was described in Materials and Methods. Mice were subjected to sham-operation or 45 min of ischemia followed by 1 week of reperfusion (I/R). Transthoracic echocardiographic analysis was performed. LVIDd, diastolic left ventricular internal diameters; LVIDs, systolic left ventricular internal diameters; FS, fractional shortening of left ventricular diameter. n = 8, *p<0.05.
(PDF)

Figure S6 Schematic model of MDRL regulation of mitochondrial fission and apoptosis via the miR-361/miR-484 pathway.
(PDF)

Table S1 Microarray analysis of miRNAs induced by Anoxia/Reoxygenation in nucleus.
(DOC)

Text S1 Supplemental methods. Additional Experimental Procedures are described in the Text S1.
(DOC)
