# Supplementary figures and images for: MDRL lncRNA Regulates the Processing of miR-484 Primary Transcript by Targeting miR-361
Source: PLoS Genet. 2014 Jul 24;10(7):e1004467. doi: 10.1371/journal.pgen.1004467 (PMC4109843; doi:10.1371/journal.pgen.1004467)

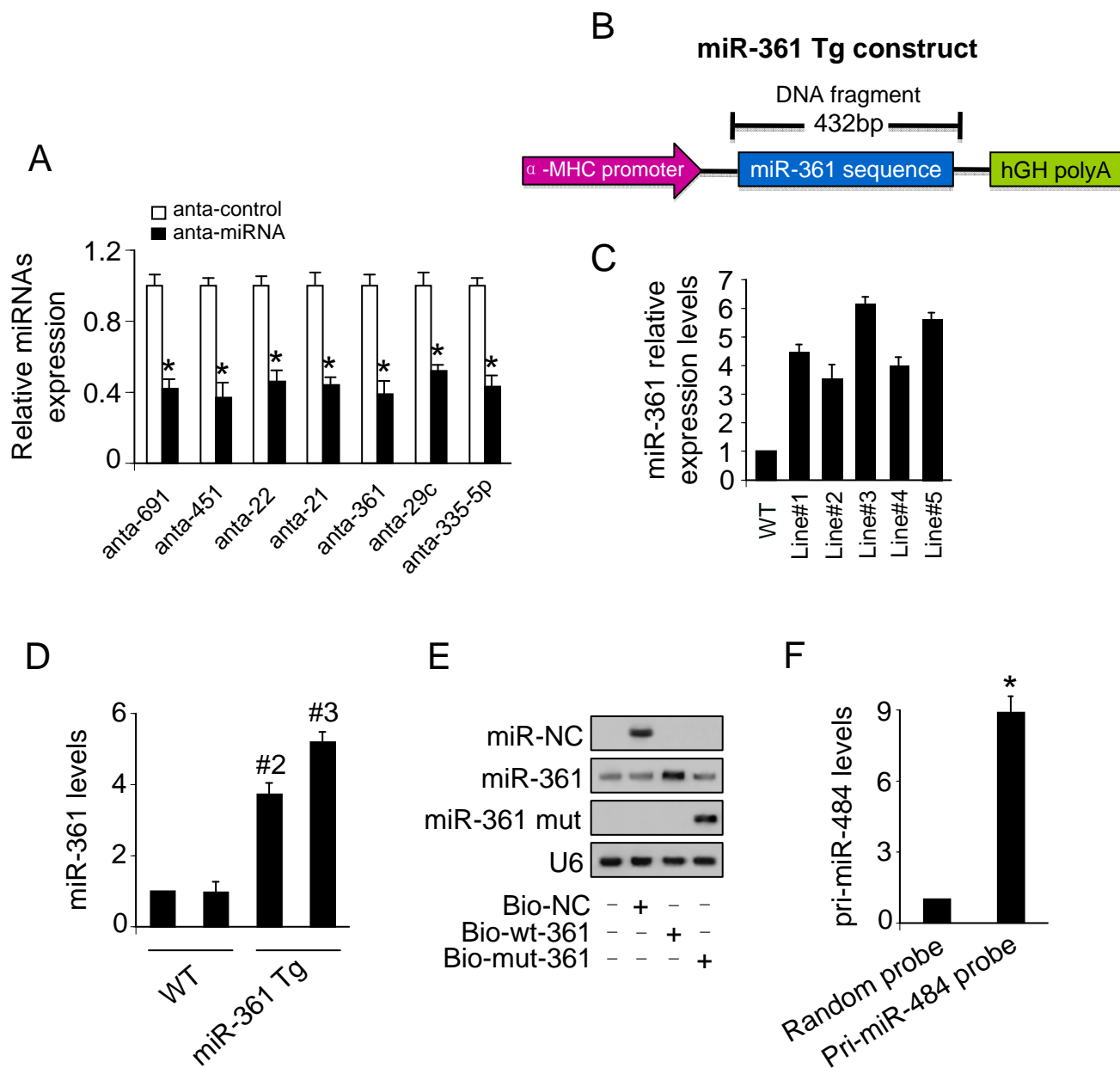

Supplement: Figure S1 — miR-361 transgenic mice genotyping assay. A. miRNAs levels are knocked down by transfecting cardiomyocytes with antagomir. Cardiomyocytes were transfected with indicated miRNA antagomir (anta-miRNA) or the antagomir control (anta-control). 48 h after transfection, the expression of miRNAs were analyzed by qRT-PCR. B. Schematic map showing the construct of miR-361 transgenic mice. C. The expression of miR-361 was analyzed by qRT-PCR from wild type and miR-361 transgenic mice of different lines. The results were normalized to U6. D. miR-361 levels analysis. miR-361 levels in the hearts of WT and miR-361 transgenic mice were analyzed by qRT-PCR. E. The transfection efficiency of miR-361. Cardiomyocytes were transfected with biotinylated wild type miR-361 (Bio-wt-361), biotinylated mutant miR-361 (Bio-mut-361) and biotinylated negative control (Bio-NC). The expression levels of miR-NC, miR-361 and miR-361 mut were analyzed by northern blot. F. pri-miR-484 levels assay. pri-miR-484 probe-coated magnetic bead was incubated with cardiomyocyte nulear lysate. After washing and enrichment of beads/RNA complex, RNA was eluted from the streptavidin beads and pri-miR-484 levels were analyzed by qRT-PCR. *p<0.05 vs Random probe. (PDF) [file pgen.1004467.s001.pdf]

A

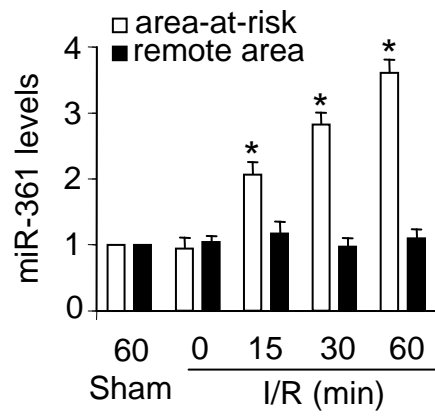

B

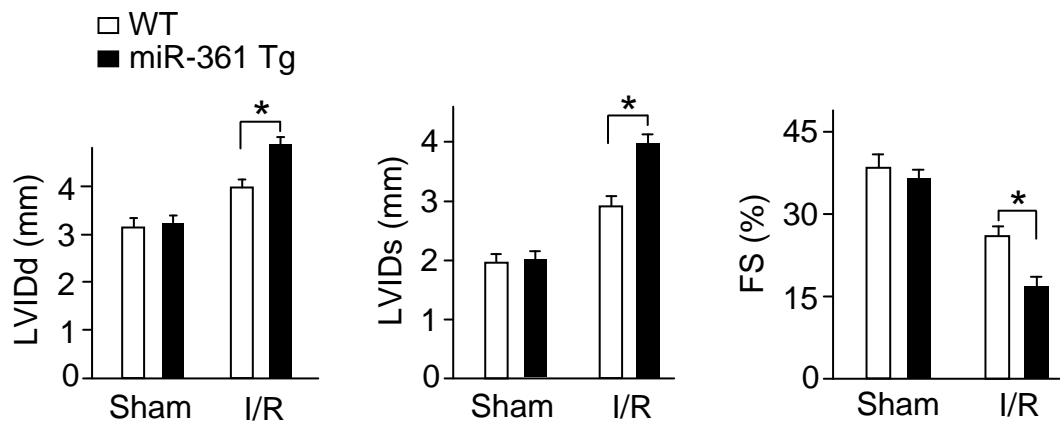

Supplement: Figure S2 — miR-361 is upregulated upon ischemia/reperfusion. A. miR-361 levels during myocardial ischemia/reperfusion. Mice were induced to undergo cardiac ischemia/reperfusion. Area-at-risk and the remote area were prepared at the indicated time for qRT-PCR analysis of miR-361 levels. n = 7, *p<0.05 vs 0 min or sham. B. miR-361 transgenic mice exhibit more severe cardiac dysfunction upon I/R. Mice were treated as described above. Transthoracic echocardiographic analysis was performed at 1 week after sham or I/R. LVIDd, diastolic left ventricular internal diameters; LVIDs, systolic left ventricular internal diameters; FS, fractional shortening of left ventricular diameter. n = 8, *p<0.05. (PDF) [file pgen.1004467.s002.pdf]

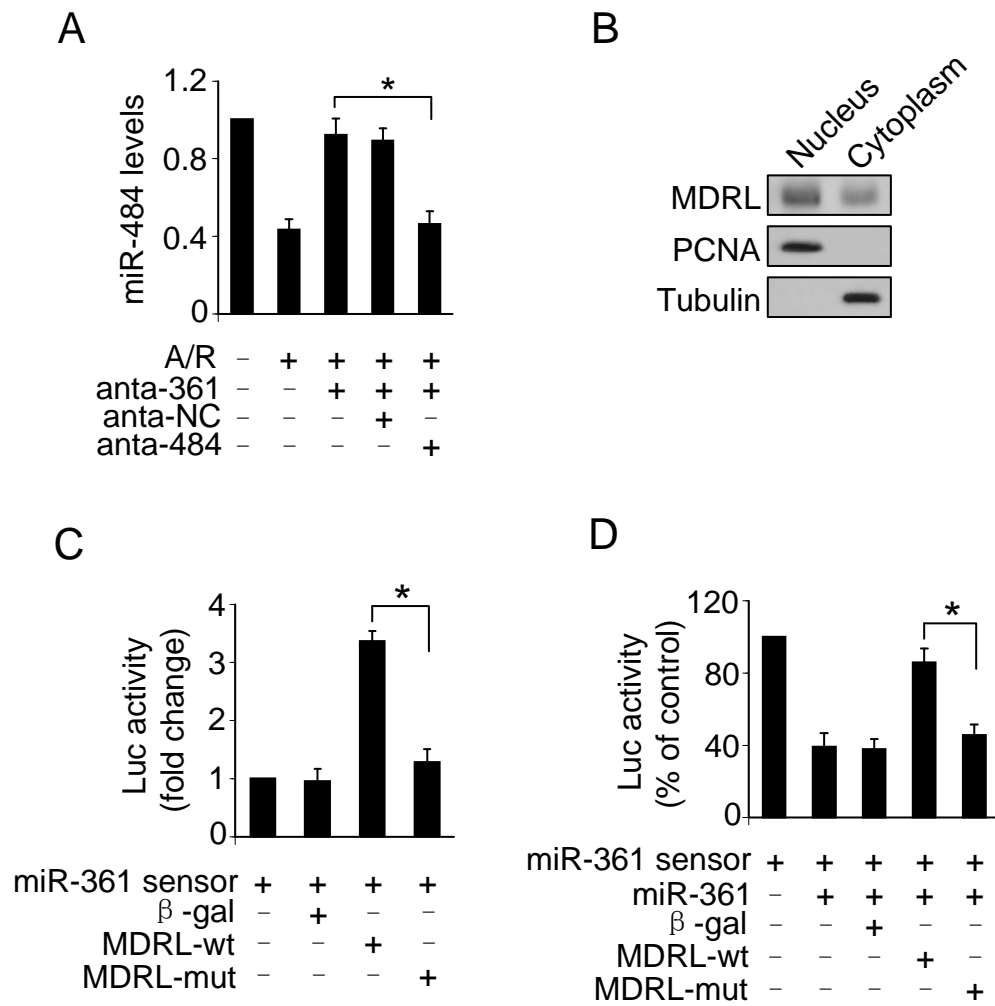

Supplement: Figure S3 — MDRL regulates miR-361 activity. A. miR-484 levels analysis. Cardiomyocytes were transfeted with the miR-361 antagomir, miR-484 antagomir or antagomir negative control, and then treated with A/R. miR-484 levels were analyzed by qRT-PCR. *p<0.05. B. Detection of MDRL in nuclear or cytoplasmic fractions in cardiomyocytes. The levels of MDRL were analyzed by northern blot. C. MDRL reduces miR-361 activity. Cardiomyocytes were infected with adenoviral MDRL-wt, MDRL-mut or β-gal, then transfected with miR-361 sensor. Luciferase activity was analyzed. *p<0.05. D. MDRL acts as a sponge for miR-361 activity. Cardiomyocytes were infected with adenoviral miR-361, MDRL-wt, MDRL-mut or β-gal, then transfected with miR-361 sensor. Luciferase activity was analyzed. *p<0.05. (PDF) [file pgen.1004467.s003.pdf]

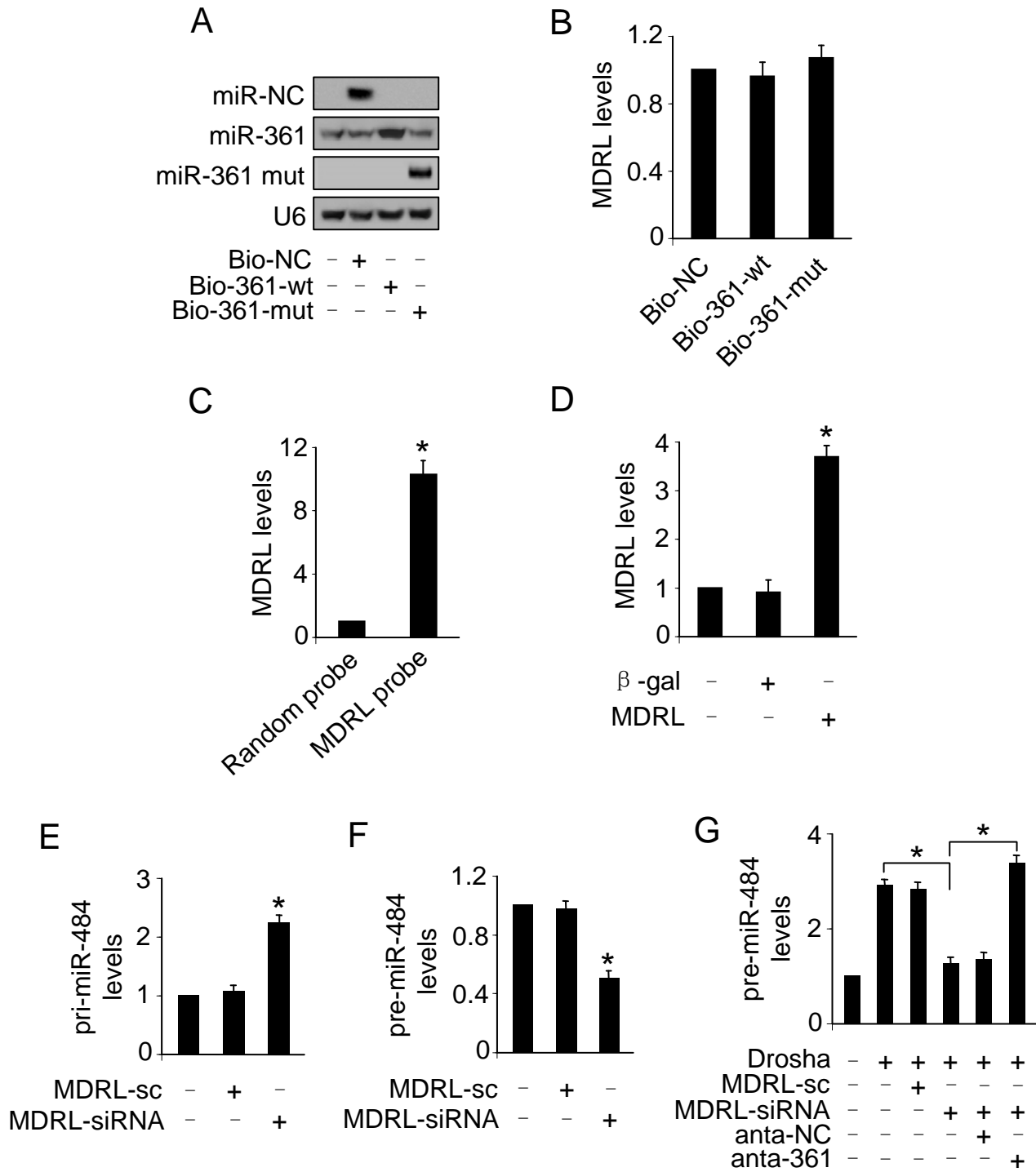

Supplement: Figure S4 — MDRL regulates the processing of pri-miR-484. A. The transfection efficiency of miR-361. Cardiomyocytes were transfected with biotinylated wild type miR-361 (Bio-361-wt), biotinylated mutant miR-361 (Bio-361-mut) and biotinylated negative control (Bio-NC). The expression levels of miR-NC, miR-361 and miR-361 mut were analyzed by northern blot. B. Cardiomyocytes were treated as described above. The expression levels of MDRL were analyzed by qRT-PCR. C. Cardiomyocyte nuclear lysate was incubated with MDRL probe or random probe-coated magnetic bead. After washing and enrichment of beads/RNA complex, RNA was eluted from the streptavidin beads and MDRL was analyzed by qRT-PCR. *p<0.05 vs Random probe. D. The expression levels of MDRL. Cardiomyocytes were infected with adenoviral MDRL or β-gal. MDRL levels were analyzed by qRT-PCR. *p<0.05 vs control. E and F. Knockdown of MDRL led to the accumulation of pri-miR-484 and the reduction of pre-miR-484 levels. Cardiomyocytes were infected with adenoviral MDRL-siRNA or MDRL-sc, the expression of pri-miR-484 (E) and pre-miR-484 (F) were analyzed by qRT-PCR. *p<0.05 vs control. G. Knockdown of miR-361 attenuated the inhibitory effects of MDRL knockdown on the processing of pri-miR-484 induced by Drosha. Cardiomyocytes were coinfected with the adenoviral Drosha, MDRL-siRNA and MDRL-sc, transfected with anta-361 or anta-NC. 48 h after transfection, cells were harvested. pre-miR-484 expression levels were analyzed by qRT-PCR. *p<0.05. (PDF) [file pgen.1004467.s004.pdf]

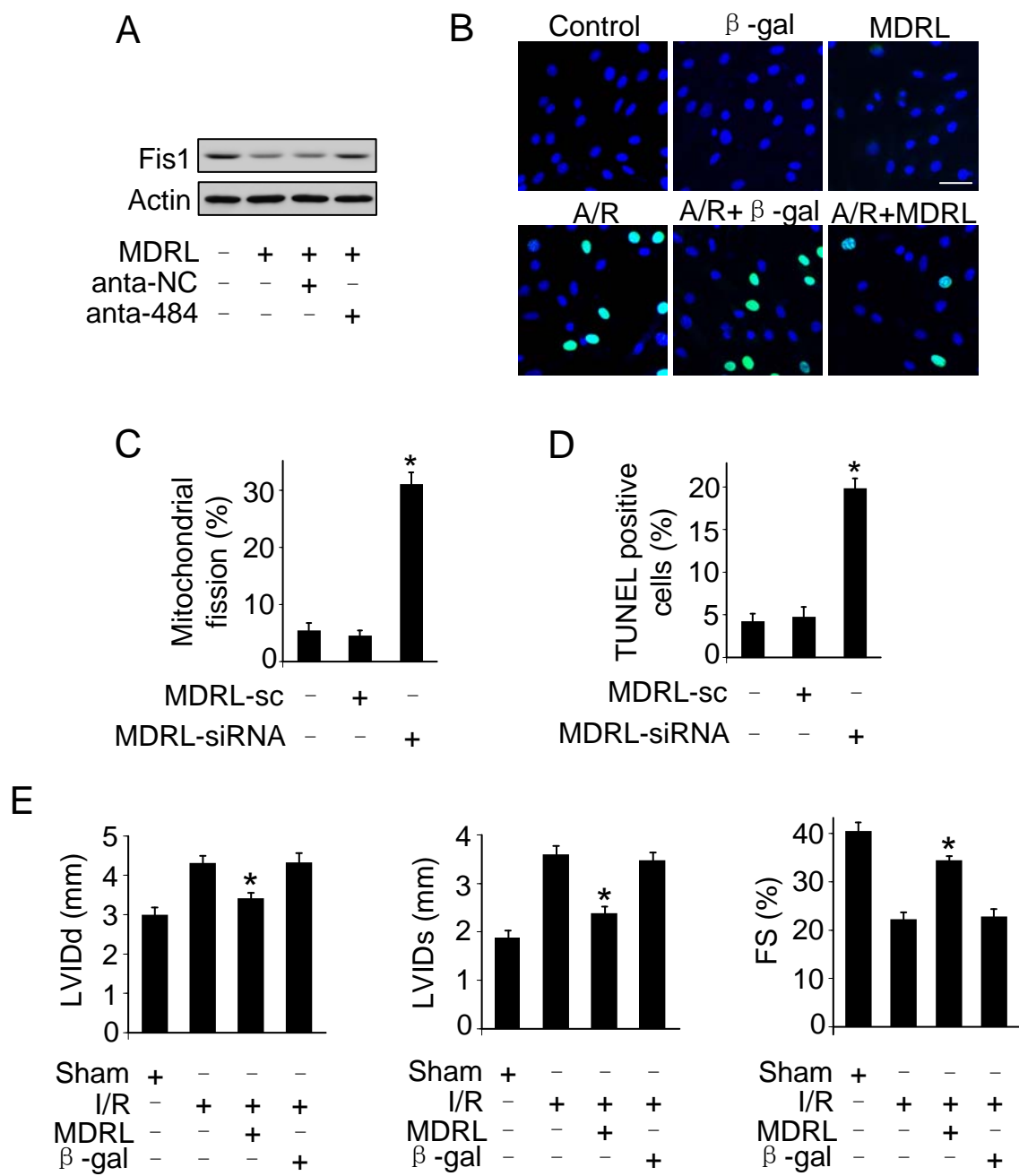

Supplement: Figure S5 — Knockdown of MDRL induces mitochondrial fission and apoptosis. A. MDRL regulates Fis1 expression by miR-484. Cardiomyocytes were infected with the adenoviral MDRL, miR-484 antagomir or antagomir-NC. The expression levels of Fis1 were analyzed by immunoblot. B. MDRL inhibits apoptosis induced by A/R. Cardiomyocytes were infected with adenoviral MDRL or β-gal, and then were exposed to A/R. TUNEL was employed to analyze apoptotic cells. Bar = 50 µm. C and D. Knockdown of MDRL promotes mitochondrial fission and apoptosis. Cardiomyocytes were infected with adenoviral MDRL-siRNA or MDRL-sc. Mitochondrial fission (C) and apoptosis (D) were analyzed. E. Intracoronary delivery of adenoviral constructs of MDRL or β-gal to the hearts was described in Materials and Methods. Mice were subjected to sham-operation or 45 min of ischemia followed by 1 week of reperfusion (I/R). Transthoracic echocardiographic analysis was performed. LVIDd, diastolic left ventricular internal diameters; LVIDs, systolic left ventricular internal diameters; FS, fractional shortening of left ventricular diameter. n = 8, *p<0.05. (PDF) [file pgen.1004467.s005.pdf]

Supplementary Fig. 6

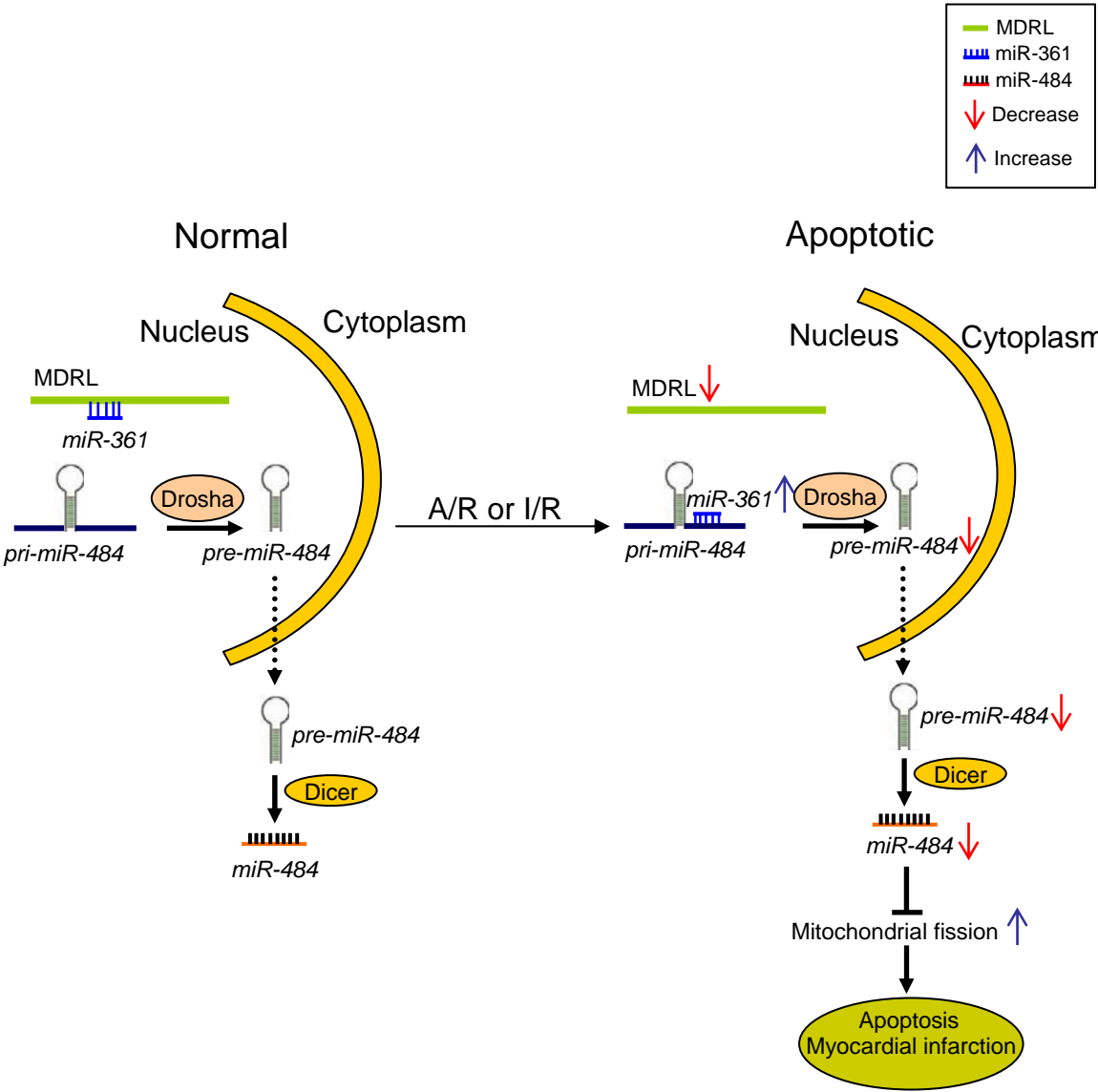

Supplement: Figure S6 — Schematic model of MDRL regulation of mitochondrial fission and apoptosis via the miR-361/miR-484 pathway. (PDF) [file pgen.1004467.s006.pdf]
