# Supplementary material for: MDRL lncRNA Regulates the Processing of miR-484 Primary Transcript by Targeting miR-361
Source: PLoS Genet. 2014 Jul 24;10(7):e1004467. doi: 10.1371/journal.pgen.1004467 (PMC4109843; doi:10.1371/journal.pgen.1004467)
Supplement: Table S1 — Microarray analysis of miRNAs induced by Anoxia/Reoxygenation in nucleus. (DOC) [file pgen.1004467.s007.doc]

**Table S1. Microarray analysis of miRNAs induced by Anoxia/Reoxygenation in nucleus.**

| **ProbeSetID** | **Average intensities** | | **Fold change**  **(A/R/control)** |
| --- | --- | --- | --- |
|  | **Control** | **A/R** |  |
| **Upregulated genes** | | | |
| mmu-miR-361_st | 39.81655 | 94.19292 | 2.8657 |
| mmu-miR-21_st | 42.20953 | 93.12211 | 2.1262 |
| mmu-miR-22-star_st | 42.01955 | 88.39046 | 2.1036 |
| mmu-miR-335-5p_st | 21.98939 | 45.14235 | 2.0529 |
| mmu-miR-29c_st | 57.1003 | 117.1298 | 2.0513 |
| mmu-miR-451_st | 93.21705 | 180.8304 | 1.9399 |
| mmu-miR-691_st | 75.32278 | 138.4051 | 1.8375 |
| mmu-miR-199b_st | 530.1528 | 956.7749 | 1.8047 |
| mmu-miR-19b_st | 303.5055 | 541.1324 | 1.7829 |
| mmu-miR-29b_st | 40.09371 | 71.0029 | 1.7709 |
| **Downregulated genes** | | | |
| mmu-miR-214_st | 616.3263 | 384.2129 | 0.6234 |
| mmu-miR-193b_st | 178.0905 | 110.3927 | 0.6199 |
| mmu-miR-149_st | 1129.596 | 696.7084 | 0.6168 |
| mmu-miR-574-5p_st | 919.0909 | 559.072 | 0.6083 |
| mmu-miR-486_st | 2083.182 | 1245.985 | 0.5981 |
| mmu-miR-16-star_st | 496.5165 | 295.2285 | 0.5946 |
| mmu-miR-140-star_st | 928.125 | 550.1912 | 0.5928 |
| mmu-miR-99b_st | 801.9457 | 474.4167 | 0.5916 |
| mmu-miR-1196_st | 1658.999 | 872.1121 | 0.5257 |
| mmu-miR-322_st | 447.0291 | 187.5734 | 0.4196 |
