# Supplementary material for: MDRL lncRNA Regulates the Processing of miR-484 Primary Transcript by Targeting miR-361
Source: PLoS Genet. 2014 Jul 24;10(7):e1004467. doi: 10.1371/journal.pgen.1004467 (PMC4109843; doi:10.1371/journal.pgen.1004467)
Supplement: Text S1 — Supplemental methods. Additional Experimental Procedures are described in the Text S1. (DOC) [file pgen.1004467.s008.doc]

**Supplementary Methods**

**Apoptosis assays**

Apoptosis was determined by the terminal deoxyribonucleotidyl transferase–mediated TUNEL using a kit from Roche. The detection procedures were in accordance with the kit instructions.

**Transfection of the antagomirs**

The chemically modified antagomirs complementary to miR-361 and miR-484 designed to inhibit endogenous miR-361 and miR-484 expression, the antagomir negative control (antagomir-NC) were obtained from GenePharma Co. Ltd. The miR-361 antagomir sequence was 5’-GUACCCCUGGAGAUUCUGAUAA-3’; the miR-484 antagomir sequence was 5’-AUCGGGAGGGGACUGAGCCUGA-3’. The antagomir-NC sequence was 5’-CAGUACUUUUGUGUAGUACAA-3’. Cells were transfected with the antagomirs or the antagomir-NC using Lipofectamine 2000 (Invitrogen) according to the manufacturer's instruction.

**Preparations of miR-361 expression constructs**

miR-361 was synthesized by PCR using mouse genomic DNA as the template. The upstream primer was 5’-CTGAGGGAAAACAAATCTTACC-3’; the downstream primer was 5’-CAGGTGTTACAGCATTAGAAAG-3’. The PCR fragment was finally cloned into the Adeno-X^TM^ Expression System (Clontech) according to the manufacturer's instructions.

**Adenoviral constructions and infection**

The mouse MDRL and mouse Drosha were synthesized by PCR using mouse cDNA as the template. The adenoviruses harboring the MDRL and Drosha were constructed using the Adeno-X^TM^ expression system (Clontech). The adenovirus containing β-galactosidase (β-gal) is as we described elsewhere . The mouse MDRL RNA interference (siRNA) target sequence is 5’-CCTGAGCCCTGAATGCAGA-3’. A scramble form was used as a control, 5’-GGACTCAGCCAGTCGACTA-3’. The adenoviruses harboring MDRL siRNA or its scramble form were constructed using the pSilencer™ adeno 1.0-CMV System (Ambion) according to the Kit’s instructions. All constructs were amplified in HEK293 cells. Adenoviral infection of cardiomyocytes was performed as we described previously .

**Preparations of subcellular fractions**

Subcellular fractions were prepared as we described . In brief, the cells were washed twice with PBS and the pellets were suspended in 0.2 ml of buffer A (20 mmol/L HEPES pH 7.5, 10 mmol/L KCl, 1.5 mmol/L MgCl_2_, 1 mmol/L EGTA, 1 mmol/L EDTA, 1 mmol/L DTT, 0.1 mmol/L PMSF, 250 mmol/L sucrose) containing a protease inhibitor cocktail. The cells were homogenized by 12 strokes in a Dounce homogenizer. The homogenates were centrifuged twice at 750 g for 5 min at 4 °C to collect nuclei and debris. The supernatants were centrifuged at 10000 g for 15 min at 4°C to collect mitochondria-enriched heavy membrane (HM) pellet. The resulting supernatants were centrifuged to yield cytosolic fractions.

**Immunoblot**

Immunoblot was performed as we described . In brief, cells were lysed for 1 h at 4°C in a lysis buffer (20 mM Tris [pH 7.5], 2 mM EDTA, 3 mM EGTA, 2 mM DTT, 250 mM sucrose, 0.1 mM PMSF, 1% Triton X-100 and a protease inhibitor cocktail). Samples were subjected to 12% SDS-PAGE and transferred to nitrocellulose membranes. Equal-protein loading was controlled by Ponceau red staining of membranes. Blots were probed using antibodies. The Fis1 antibody and PCNA antibody were from Santa Cruz Biotechnology, the Tubulin antibody was from Abcam, and horseradish peroxidase-conjugated goat anti-rabbit or rabbit anti-goat IgG were purchased from Santa Cruz Biotechnology.

**Quantitative reverse transcription-PCR (qRT-PCR)**

Stem-loop qRT-PCR for mature miR-361 was performed as described on an Applied Biosystems AB 7000 Real Time PCR system . Total RNA was extracted using Trizol reagent. After DNAse I (Takara, Japan) treatment, RNA was reverse transcribed with reverse transcriptase (ReverTra Ace, Toyobo). The levels of miR-361 analyzed by qRT-PCR were normalized to that of U6. U6 primers were forward: 5’-GCTTCGGCAGCACATATACTAA-3’; reverse: 5’-AACGCTTCACGAATTTGCGT-3’. qRT-PCR for pri-miR-484 and pre-miR-484 were performed. The sequences of pri-miR-484 primers were forward: 5’-GGCGGGGCCTCGCGGCCCTG-3’; reverse: 5’-TACGCCGCCAGCCCCCTAGG-3’; the sequences of pre-miR-484 primers were forward: 5’-CTCGTCAGGCTCAGTCCCCT-3’; reverse: 5’-TACGCCGCCAGCCCCCTAGG-3’. qRT-PCR for MDRL were performed as we described . The sequences of MDRL primers were forward: 5’-CTCCTGCGCTTTAATTTCCAA-3’; reverse: 5’-TGGCTCTTCCGTAGTTGATGA-3’. The results were standardized to control values of glyceraldehyde-3-phosphate dehydrogenase (GAPDH). GAPDH forward primer: 5’-TGTGTCCGTCGTGGATCTGA-3’; reverse: 5’-CCTGCTTCACCACCTTCTTGA-3’. The specificity of the PCR amplification was confirmed by agarose gel electrophoresis.

**Northern blot analysis**

Northern blot was performed as described . In brief, the samples were run on a 15% polyacrylamide-urea gel, transferred to positively charged nylon membranes (Millipore) followed by cross-linking through UV irradiation. The membranes were subjected to hybridization with 100 pmol 3’-digoxigenin (DIG)-labeled probes overnight at 42°C. Probes were labeled with DIG using a 3’-End DIG Labeling Kit (Roche). The detection was performed using a DIG luminescent detection kit (MyLab) according to the manufacturer’s instructions. The probe sequence for miR-361 was 5’-GTACCCCTGGAGATTCTGATAA-3’. The probe sequence for miR-361 mutant was 5’-GTACCCCTGGAGATTCTTTGTC-3’. The probe sequence for miR-484 was 5’-ATCGGGAGGGGACTGAGCCTGA-3’. The probe sequence for miR-NC was 5’-TTGTACTACACAAAAGTACTG-3’. DIG-labeled U6 probe was used as an internal control, and its sequence was 5’-TGGAACGCTTCACGAATTTG-3’.

**Microarray analysis**

Total RNA was extracted from cardiomyocytes by using Trizol reagent. For the miRNA microarray experiments, low-molecular-weight RNA was isolated with the described method , and then used for miRNA microarray by using the Affymetrix Arrays (CapitalBio Corp.). 20 miRNAs were identified to have at least a 1.5 fold change in expression.

**Luciferase constructs and transfection of MDRL and miR-361 sensor reporter**

Mouse MDRL wild type (MDRL-wt) and the mutant (MDRL-mut) were cloned downstream the coding region of luciferase gene. The forward primer was 5’-AGGCAGTGCTGTCTGGGCAAA-3’; the reverse primer was 5’-TGGCTCTTCCGTAGTTGATGA-3’. miR-361 sensor reporter was constructed according to the method previously described . Briefly, mouse genomic sequence (200bp) flanking pre-miR-361 was reversely inserted into the pGL3 vector, downstream of the coding region of luciferase gene.

Cardiomyocytes were infected with the indicated adenoviruses, then transfected with the indicated luciferase constructs as described in the corresponding figure legends. The transfection was performed using Lipofectamine 2000 (Invitrogen) according to the manufacturer's instruction. The luciferase activity was analyzed as we described elsewhere .

**miRNA pull-down assay with biotinylated miRNA**

Cardiomyocytes were transfected with biotinylated miRNAs (50 nM), harvested 72h after transfection. The cells were washed with PBS followed by brief vortex, and incubated in a lysis buffer [20 mM Tris, pH 7.5, 200 mM NaCl, 2.5 mM MgCl_2_, 0.05% Igepal, 60 U/mL Superase-In (Ambion), 1 mM DTT, protease inhibitors (Roche)] on ice for 10 min. The lysates were precleared by centrifugation, and 50μl of the samples were aliquoted for input. The remaining lysates were incubated with M-280 Streptaviden magnetic beads (Sigma). To prevent non-specific binding of RNA and protein complexes, the beads were coated with RNase-free BSA and yeast tRNA (both from Sigma). The beads were incubated at 4℃ for 3h, washed twice with ice-cold lysis buffer, three times with the low salt buffer (0.1%SDS, 1%Trition X-100, 2 mM EDTA, 20 mM Tris-HCl pH8.0, 150 mM NaCl), and once with the high salt buffer (0.1%SDS, 1%Trition X-100, 2 mM EDTA, 20 mM Tris-HCl pH 8.0, 500 mM NaCl). The bound RNAs were purified using Trizol for the analysis.

**Pull-down assay with biotinylated DNA probe**

The biotinylated DNA probe complementary to MDRL was synthesized and dissolved in 500 μl of wash/binding buffer (0.5M NaCl, 20 mM Tris-HCl, pH 7.5, and 1 mM EDTA). The probes were incubated with streptavidin-coated magnetic beads (Sigma) at 25 °C for 2 h to generate probe-coated magnetic beads. Cardiomyocyte lysates were incubated with probe-coated beads, and after washing with the wash/binding buffer, the RNA complexes bound to the beads were eluted and extracted for Northern blot analysis. The following primer sequences were used: MDRL pull-down probe, 5’-GCCCAGATGCGCGTGTTCTCTTCCC-3’; and random pull-down probe, 5’-TGATGTCTAGCGCTTGGGCTTTG-3’.

**Intracoronary delivery of adenoviruses, ischemia/reperfusion (I/R), preparations of area-at-risk, and miR-361 antagomir delivery**

Male adult C57BL/6 mice (8 weeks old) were obtained from Institute of Laboratory Animal Science of Chinese Academy of Medical Sciences (Beijing, China). All experiments were performed according to the protocols approved by the Institute Animal Care Committee. The mice were anesthetized and ventilated with a HX-300S animal ventilator. The chest was entered through a small left anterior thoracotomy, the pericardial sac was then removed, and 2×10^11^ moi adenoviruses of MDRL were injected with a catheter from the apex of the left ventricle into the aortic root while the aorta and pulmonary arteries were cross-clamped. The clamp was maintained for 20s when the heart pumped against a closed system. After removal of air and blood, the chest was closed with 8-0 silk suture and the animal was extubated and transferred back to its cage. The heart was monitored by electrocardiography through the experimental period. Five days after the injection of adenoviruses, the mice were re-anesthetized and ventilated. The chest was reopened and the heart was exposed to identify the left anterior descending coronary artery (LAD). A 8-0 silk suture was passed around the LAD at the inferior border of left auricle and the artery was occluded by snaring with a vinyl tube through which the ligature had been passed. The coronary artery was occluded by pulling the snare tight and securing it with a hemostat. After 45min ischemia, the ligature was released and the heart was reperfused. Sham-operated group experienced the same procedure except the snare was left untied. After 24 hours of reperfusion, the mice were anesthetized. The thoracotomy was reopened and the suture was re-ligated. Evans blue dye (1 ml of a 2.0% solution; Sigma-Aldrich) was injected into jugular vein into the heart for delineation of the ischemic zone from the nonischemic zone. The heart was rapidly excised. The heart slices were incubated in 1.0% 2,3,5-triphenyltetrazolium chloride (Sigma-Aldrich) for 15 minutes at 37°C for demarcation of the viable and nonviable myocardium within the risk zone. The staining was stopped by ice-cold sterile saline and the slices were fixed in 10% neutral buffered formaldehyde and individually weighed. Both sides of each slice were photographed. Each of the myocardial slices was weighed and the areas of infarction (INF), area at risk (AAR), and nonischemic left ventricle (LV) were assessed with computer-assisted planimetry (NIH Image 1.57) by an observer blinded to sample identity. The ratio of AAR/LV, INF/AAR and INF/LV were calculated. AAR in the center of the territory of the left anterior descending coronary artery and the remote area in the posterior part of the left ventricle far from the AAR were prepared as described .

Chemically modified antagomirs were 2’-OMe modified (GenePharma，Shanghai). The mice received on three consecutive days, intravenous injections of antagomirs and their controls at doses of 35 mg/kg body weight in a small volume (0.2 ml) per injection.

**Echocardiographic assessment**

Transthoracic echocardiographic analysis was performed on mice after 1 week of the sham or I/R surgery as we described . Echocardiographic parameters such as systolic left ventricular internal diameters (LVIDs) and diastolic left ventricular internal diameters (LVIDd) were measured. Fractional shortening (FS) of left ventricular diameter was shown. After in vivo evaluation of cardiac function the mice were euthanized and the hearts were harvested, weighted and used for histological examination.

**Histology**

The harvested hearts were fixed in 10% formalin and embedded in paraffin and sectioned at 6μm thickness. TUNEL staining was performed as manufacture’s procedure (Roche). Cardiomyocytes were identified by α-actinin and the total nuclei were stained by DAPI. Magnification ×40 photos were taken and 25 random fields for each heart sample were quantified by an investigator who was blind to the treatment.

**Electron Microscopy**

Heart ultrastructural analyses were performed to quantify mitochondrial fission. Sample preparations and conventional EM were carried out as described before . Samples were examined at a magnification of 15,000 with a JEOL JEM-1230 transmission electron microscope. For comparison of mitochondrial fission, EM micrographs of thin sections were evaluated. The size of individual mitochondrion was measured by using Image-Pro Plus software. Approximately 1000-1500 mitochondria were measured to determine the percentages of mitochondria with various sizes. In I/R treated heart tissues, mitochondria disintegrated into numerous small round fragments of varying size, the number of small mitochondrion was increasing. Thus, we determined the mitochondria with size less than 0.6 mm^2^ as fission mitochondria.

**References**

Chen, C., D. A. Ridzon, et al. (2005). "Real-time quantification of microRNAs by stem–loop RT–PCR." Nucleic acids research **33**(20): e179-e179.

Kim, C.-H., Y.-S. Cho, et al. (2002). "Early expression of myocardial HIF-1α in response to mechanical stresses regulation by stretch-activated channels and the phosphatidylinositol 3-kinase signaling pathway." Circulation research **90**(2): e25-e33.

Lau, P., J. D. Verrier, et al. (2008). "Identification of dynamically regulated microRNA and mRNA networks in developing oligodendrocytes." The Journal of Neuroscience **28**(45): 11720-11730.

Li, P.-F., J. Li, et al. (2002). "Phosphorylation by protein kinase CK2: a signaling switch for the caspase-inhibiting protein ARC." Molecular cell **10**(2): 247-258.

Li, Y.-Z., D.-Y. Lu, et al. (2008). "p53 initiates apoptosis by transcriptionally targeting the antiapoptotic protein ARC." Molecular and cellular biology **28**(2): 564-574.

Lin, Z., I. Murtaza, et al. (2009). "miR-23a functions downstream of NFATc3 to regulate cardiac hypertrophy." Proceedings of the National Academy of Sciences **106**(29): 12103-12108.

Murtaza, I., H.-X. Wang, et al. (2008). "Down-regulation of catalase and oxidative modification of protein kinase CK2 lead to the failure of apoptosis repressor with caspase recruitment domain to inhibit cardiomyocyte hypertrophy." Journal of biological chemistry **283**(10): 5996-6004.

Tan, W.-Q., K. Wang, et al. (2008). "Foxo3a inhibits cardiomyocyte hypertrophy through transactivating catalase." Journal of biological chemistry **283**(44): 29730-29739.

Thomson, J. M., J. Parker, et al. (2004). "A custom microarray platform for analysis of microRNA gene expression." Nature Methods **1**(1): 47-53.

Wang, J.-X., J.-Q. Jiao, et al. (2011). "miR-499 regulates mitochondrial dynamics by targeting calcineurin and dynamin-related protein-1." Nature medicine **17**(1): 71-78.

Wang, J., X. Liu, et al. (2010). "CREB up-regulates long non-coding RNA, HULC expression through interaction with microRNA-372 in liver cancer." Nucleic acids research **38**(16): 5366-5383.

Wang, K., B. Long, et al. (2012). "miR-484 regulates mitochondrial network through targeting Fis1." Nature communications **3**: 781.
